# Sex Classification Based on the Functional Connectivity Patterns of the Language Network: A Resting State fMRI Study

**DOI:** 10.1002/hbm.70450

**Published:** 2026-01-10

**Authors:** X. Lajoie, C. DeRoy, C. Bedetti, B. Houzé, N. Clarke, S. Hétu, M.‐È. Picard, L. Bellec, S. M. Brambati

**Affiliations:** ^1^ Centre de Recherche de l’Institut Universitaire de Gériatrie de Montréal Montréal Québec Canada; ^2^ Faculté des Arts et des Sciences, Département de Psychologie Université de Montréal, H2V 2S9 Montréal Québec Canada; ^3^ Centre Interdisciplinaire de Recherche sur le Cerveau et l’Apprentissage Montréal Québec Canada

**Keywords:** language network, machine learning, resting‐state functional connectivity, sex classification

## Abstract

Research on sex differences in the brain is essential for a better understanding of how the brain develops and ages, and how neurological and psychiatric conditions can impact men and women differently. While numerous studies have focused on sex differences in brain structures, few have examined the characteristics of functional networks, particularly the language network. Although previous research suggests similar overall language performance across sexes, differences may still exist in the brain networks that underlie language processing. In addition, prior studies on sex differences in language have predominantly relied on task‐based fMRI, which may fail to capture subtle differences in underlying functional activity. In this study, we applied a machine learning approach to classify participants' sex based on resting‐state functional connectivity patterns of the language network in healthy young adults (270 men and 288 women; age: 22–36 years), and to identify the most predictive functional connectivity features. The classifier achieved 91.3% accuracy, with key discriminant features anchored to the left opercular part of the inferior frontal gyrus, the left planum temporale, and the left anterior middle temporal gyrus. These regions show distinctive connectivity patterns with heteromodal association cortices, including the occipital poles, angular gyrus, posterior cingulate gyrus, and intraparietal sulcus. Although there was an overlap between men and women, men displayed stronger functional connectivity values in these regions. These findings highlight sex‐related differences in functional connectivity patterns of the language network at rest, underscoring the importance of considering sex as a variable in future research on language and brain function.

## Graphical abstract

We classified the resting‐state functional connectivity patterns anchored to core regions of the language network in a large sample of healthy young adults. The results showed that these patterns contain information capable of predicting sex with an accuracy of 91.3% on the test set and 78.1% on the holdout set.

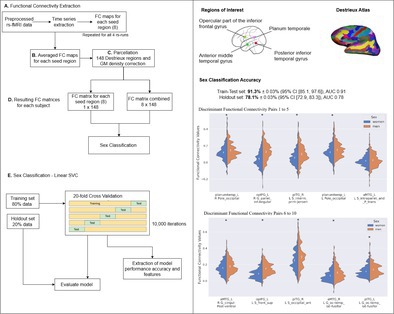

## 1 Introduction

The human brain supports a wide range of complex cognitive functions such as memory, attention, emotion, and language through the dynamic interaction of distributed neural systems. Understanding how such networks are functionally organized, and how their architecture varies across individuals, is a central goal of cognitive neuroscience. Among these, the language network plays a central role in supporting a range of communicative functions, including speech comprehension, production, phonological processing, orthographic decoding, and semantic integration (Friederici and Gierhan 2013; Price 2012). Rather than being localized to a single anatomical structure, the language network involves left lateralized and, in some cases, bilateral frontotemporal and parietal regions organized into partially segregated but interacting subsystems (Ardila et al. 2016).

Functional magnetic resonance imaging (fMRI) has been instrumental in mapping the structure and organization of this network, particularly through the analysis of functional connectivity (FC). FC is defined as the temporal coincidence of spatially distant neurophysiological events in different brain areas (Friston et al. 1993). In fMRI, this term commonly refers to the temporal correlation of blood‐oxygen‐level‐dependent (BOLD) signal fluctuations between spatially distant brain regions (Biswal et al. 1995). The assumption behind this concept is that regions displaying correlated functional behaviors are coupled or part of the same networks (Cole et al. 2014). FC provides insight into how brain regions interact as a part of a coherent network. By using either task‐based or resting‐state fMRI (rs‐fMRI), researchers can assess the FC of brain systems under different conditions (Friston 1994; Friston et al. 1997), including within the language network itself (Branco et al. 2016, 2020).

Understanding how the language network is functionally organized and how its connectivity patterns vary across individuals is a key question in cognitive neuroscience. One dimension of individual variability that remains poorly understood is the role of biological sex in shaping the architecture of the language network. While overall language performance tends to be similar between men and women, research has consistently reported subtle group‐level differences in specific language abilities such as verbal fluency, reading, and language acquisition speed, though these effects are often small and vary by age and task (Wallentin 2009). Beyond behavioral performance, sex differences in the prevalence and clinical profiles of language‐related disorders further suggest that the neural architecture supporting language may differ between sexes. For instance, voice, speech, and language disorders are more prevalent in boys than girls (Black et al. 2015; Lange et al. 2016). In older adults, studies have shown that the risk of Broca's aphasia is higher in women than in men, whereas the risk of anomic aphasia is higher in men than in women (Li et al. 2024). Additionally, men tend to exhibit more severe aphasia symptoms than women (Sharma et al. 2019). These findings raise the possibility that biological sex may influence the network‐level organization of the language network, making each sex differently vulnerable to various pathologies.

To investigate sex differences in the language functional network, prior studies have used task‐based fMRI (tb‐fMRI) to compare activation patterns between sexes during the execution of language tasks. A recent review and meta‐analysis summarizing these studies suggested that, while men and women share substantial similarities in brain activation patterns during language processing, subtle sex differences still exist in specific language‐related regions (Sato 2020). However, the exact locations of these activation differences are inconsistent across studies, resulting in inconclusive evidence of sex differences in language‐related activation patterns (Sato 2020). Moreover, task‐based analyses focus on region‐specific activation, providing limited insight into network‐level interactions. As a result, FC has emerged as a complementary approach to investigating the neural correlates of sex differences in language processing.

A notable example comes from Xu et al. (2020), who examined task‐based FC during a semantic decision task to classify participants' sex using a machine learning (ML) approach. In ML classification, the classifier model learns a possible relationship between a set of input features based on the input data (e.g., FC profile patterns) and output data labels (e.g., sex) and uses these learnings to predict sex output data labels in a test sample composed of unseen subject data. Successful sex classification based on FC patterns suggests that FC features contain sex‐specific information. The importance of ML in this context lies in its ability to model complex, high‐dimensional relationships across large FC matrices and allows the identification of relationships that may be too subtle or distributed to detect using standard statistical comparisons between groups. ML does not rely on predefined hypotheses about specific brain regions or connections but instead allows a data‐driven discovery of predictive features. The findings of Xu et al. (2020) underscore the potential of analyzing FC patterns and leveraging ML techniques as insightful tools for examining the relationship between sex and the characteristics of the language functional brain network. Although men and women showed comparable behavioral performance and similar activation patterns during the task, their FC profiles enabled sex classification with 74% accuracy, suggesting the presence of sex‐specific connectivity patterns within the language network. While these findings are promising, the study relied on a relatively small sample size (*n* = 58) and focused exclusively on semantic processing, limiting the analysis to connectivity within the semantic network. As a result, it is still unclear whether sex‐specific patterns are also found in other language subsystems, such as networks supporting articulatory‐phonological processing, speech perception, semantic processing, and orthography‐to‐phonology conversion, particularly under resting‐state conditions.

Resting‐state fMRI provides a unique opportunity to examine the intrinsic organization of brain networks without task demands, allowing for the simultaneous investigation of multiple language‐related subnetworks (Battistella et al. 2020). In contrast to task‐based designs, rs‐fMRI enables the investigation of baseline connectivity patterns across language subsystems involved in semantic processing, articulatory‐phonological processing, speech perception, and orthography‐to‐phonology conversion within a single scan session. Resting‐state data are particularly valuable for studying clinical populations, where task compliance may be difficult, and offer greater consistency across individuals and scanners (Finn and Todd Constable 2016). Additionally, unlike task‐based designs, which often require multiple task conditions to map different subnetworks, rs‐fMRI acquisition allows the investigation of FC in multiple subnetworks from the same dataset. In particular, using the seed‐to‐voxel approach on rs‐fMRI data, it has been shown that resting‐state FC (rs‐FC) patterns anchored to key regions known for articulatory, phonological, orthography‐to‐phonology conversion, and semantic processes overlap with the subnetworks of speech production, speech perception, orthography processing, and semantic processing obtained with task‐based fMRI (Battistella et al. 2020). These results suggest that, by using the seed‐to‐voxel approach on rs‐fMRI data, it is possible to simultaneously study the rs‐FC patterns in the main language subnetworks.

A growing number of studies now apply ML to rs‐fMRI data to classify sex based on whole‐brain FC patterns. Prior ML studies using this approach have demonstrated that sex classification based on whole‐brain connectivity can achieve accuracies between 70% and 87% (Al Zoubi et al. 2022; Zhang et al. 2018). However, such approaches face challenges related to high dimensionality, interpretability, and difficulty linking results to specific cognitive domains like language. To address these limitations, Weis et al. (2020) applied a ML algorithm on rs‐fMRI data to classify individuals according to their sex by using a parcel‐wise approach, which divides the brain into predefined regions, or “parcels,” based on anatomical or functional boundaries. For each individual parcel, the authors calculated the rs‐FC patterns between the parcel itself and all the other parcels in the brain. Then, the rs‐FC pattern for each parcel was separately entered into a sex‐classification ML model. Among the parcels that achieved the highest sex classification, those parcels belonging to the language domain, such as the left inferior frontal gyrus and the left temporal pole, were listed. Interestingly, the bilateral temporal lobes were also found as being discriminant for sex classification in studies using whole‐brain rs‐FC approaches (Al Zoubi et al. 2022; Zhang et al. 2018). While the parcel‐wise approach used by Weis et al. (2020) achieved a classification accuracy of approximately 74%, which is comparable to other whole‐brain FC studies, this performance suggests that additional sources of discriminative information remain unaccounted for. One possibility is that analyzing connectivity at the parcel level overlooks the complex interactions between language subsystems involved in semantic processing, articulatory‐phonological processing, speech perception, and orthography‐to‐phonology conversion, which together shape the functional organization of the language network. Treating the language network as an integrated system, rather than as isolated parcels, may provide a more comprehensive understanding of sex‐linked variability in resting‐state connectivity and potentially improve classification performance.

Taken together, these findings suggest that sex differences may exist at the level of connectivity within the language network, but current evidence is limited by narrow task scope, small sample sizes, and a lack of full‐network, including sub‐networks, resting‐state analyses. To our knowledge, no study to date has used a seed‐based approach to classify sex based on rs‐FC patterns anchored specifically to core regions of the language network, incorporating its multiple functional subsystems.

In the present study, we investigated whether sex can be classified based on resting‐state FC patterns within the core language network, using data from the Human Connectome Project (Van Essen et al. 2012). We applied a ML approach to assess whether sex‐specific connectivity patterns exist across four language subsystems: specifically, semantic processing, articulatory‐phonological processing, speech perception, and orthography‐to‐phonology conversion, in a large sample of healthy young adults. Our goal was to clarify whether the intrinsic organization of the language network differs by sex, and to explore the potential of rs‐FC for understanding sex‐linked variability in brain architecture.

## 2 Materials and Methods

### 2.1 Participants

The samples were obtained from the S1200 dataset of the HCP. The database was filtered to select participants with a complete resting‐state fMRI protocol (four rs‐fMRI sessions) and no quality control issues. Age filters were not applied to the database. Only right‐handed participants were selected if they had a laterality quotient of 50 or higher on the Edinburgh Handedness Inventory scale (Oldfield 1971). Although the term “gender” is used throughout the HCP, we use the term “sex” in this article because the database collected the subject's biological sex and not gender identification. These inclusion criteria resulted in a sample of 587 healthy adults (270 men and 317 women; age range: 22–36). However, there was a significant age imbalance between the sexes, necessitating further adjustments (mean age of men = 28.05, SD = 3.76, mean age of women = 29.06, SD = 3.71, *t* (585) = −3.258, *p* < 0.001; mean education of men = 14.96, SD = 1.68, mean education of women = 15.05, SD = 1.77, *t* (584) = −0.659, *p* = 0.255). To correct for this imbalance, we adjusted the sample to eliminate age differences between the groups, resulting in a final sample of 558 participants (270 men and 288 women; age range: 22–36). The two groups were matched for age and educational levels (mean age of men = 28.05, SD = 3.76, mean age of women = 28.48, SD = 3.38, *t* (556) = −0.425, *p* = 0.077; mean education of men = 14.96, SD = 1.68, mean education of women = 15.00, SD = 1.77, *t*(556) = −0.278, *p* = 0.390).

A train‐test sample with 80% of participants (*n* = 446) and a holdout sample of 20% (*n* = 112) were created for the classification analysis. These samples were randomly created using the Nilearn function sklearn.model_selection.train_test_split. The train and holdout samples did not differ in terms of proportion of men and women (train‐test sample: men/women = 211/235; holdout sample: men/women = 59/53, *χ* ^2^ (1) = 1.033, *p* = 0.309). Descriptive statistics for the training test and holdout samples by sex are presented in Table 1. No differences were observed in terms of age or educational level.

**TABLE 1:** Descriptive statistics for train‐test and holdout samples by sex.

sample type	Sex	*N*	Age (*M*, SD)	Education (*M*, SD)	*t* (age)	*p* (age)	*t* (education)	*p* (education)
Train‐Test	Men	211	27.99 (3.71)	14.95 (1.71)	−1.430	0.08	−0.234	0.41
	Women	235	28.47 (3.42)	14.99 (1.78)				
Holdout	Men	59	28.29 (3.99)	14.98 (1.58)	−0.375	0.35	−0.173	0.43
	Women	53	28.55 (3.24)	15.04 (1.75)				

### 2.2 Data Acquisition and Preprocessing

All participants underwent scanning on a customized Siemens 3T “Connectome Skyra” system located at Washington University in St. Louis. The MRI setup included a standard 32‐channel Siemens receiver head coil and a Siemens‐designed body transmission coil, specifically adapted for the compact space required by the specialized gradients of the WU‐Minn and MGH‐UCLA Connectome scanners. T1‐weighted 3D MPRAGE images were obtained using the following parameters: repetition time (TR) = 2400 ms; echo time (TE) = 2.14 ms; inversion time (TI) = 1000 ms; flip angle = 8°; field of view (FOV) = 224 × 224 mm; and voxel size = 0.7 mm isotropic. rs‐fMRI images were acquired using a gradient‐echo EPI sequence with the following parameters: TR = 720 ms; echo time (TE) = 33.1 ms; echo spacing = 0.58 ms; flip angle = 52°; FOV = 208 × 180 mm (RO × PE); voxel size = 2 × 2 × 2 mm. A total of 72 slices were acquired to cover the entire brain. Each participant underwent four resting‐state acquisition sessions performed in two sessions, with two runs per session. The data consisted of 1200 volumes for each run, for a total of 4800 volumes for each subject. For the resting‐state data acquisition, subjects were asked to lie with eyes open, with “relaxed” fixation on a white cross (on a dark background), think of nothing in particular, and to not fall asleep. Each resting‐state scan lasted approximately 14.4 min. A complete description of the protocol is provided in the HCP S1200 Release Reference Manual (Human Connectome Project 2017).

For the resting‐state data analysis, we downloaded the preprocessed data provided by HCP (S1200 release). First, the data were subjected to the HCP minimal preprocessing pipeline (Glasser et al. 2013). Pre‐processing included the following steps: (1) correction of gradient‐nonlinearity‐induced distortion; (2) realignment of the time series to correct for subject motion via rigid‐body realignment to the single‐band reference image; (3) correction for distortion in the phase‐encoding direction; (4) correction for distortions of the single‐band reference image using spline interpolation and registration to the T1w image; and (5) nonlinear registration of the rs‐fMRI data to the MNI space using one‐step spline resampling, followed by intensity normalization, brain masking, and high‐pass temporal filtering to remove slow drifts (cutoff period ~ 100.0 s, Griffanti et al. 2014). The data were denoised using Independent Component Analysis followed by FMRIB's ICA‐based X‐noiseifier (ICA‐FIX) to remove “noise components,” such as the effects of motion, scanner artefacts, and non‐neuronal physiology, including white matter signal components (Griffanti et al. 2014; Salimi‐Khorshidi et al. 2014). Additionally, the 24 head motion parameters (Satterthwaite et al. 2013) were regressed out of the data. These HCP‐preprocessed data were used to ensure high‐quality data processing and consistency across the studies. HCP‐preprocessed functional data were smoothed using an isotropic Gaussian kernel of 6 mm full width at half maximum (FWHM) with *Nilearn*'*s NiftiMasker*. Importantly, for the seed‐to‐voxel connectivity analysis, the time series for each seed region were extracted from the unsmoothed images to reduce potential signal contamination from neighboring voxels and to preserve the local specificity of the seed signals (Ely et al. 2016).

### 2.3 Seed‐to‐Voxel Analysis of Resting‐State Functional Connectivity

**FIGURE 1:**
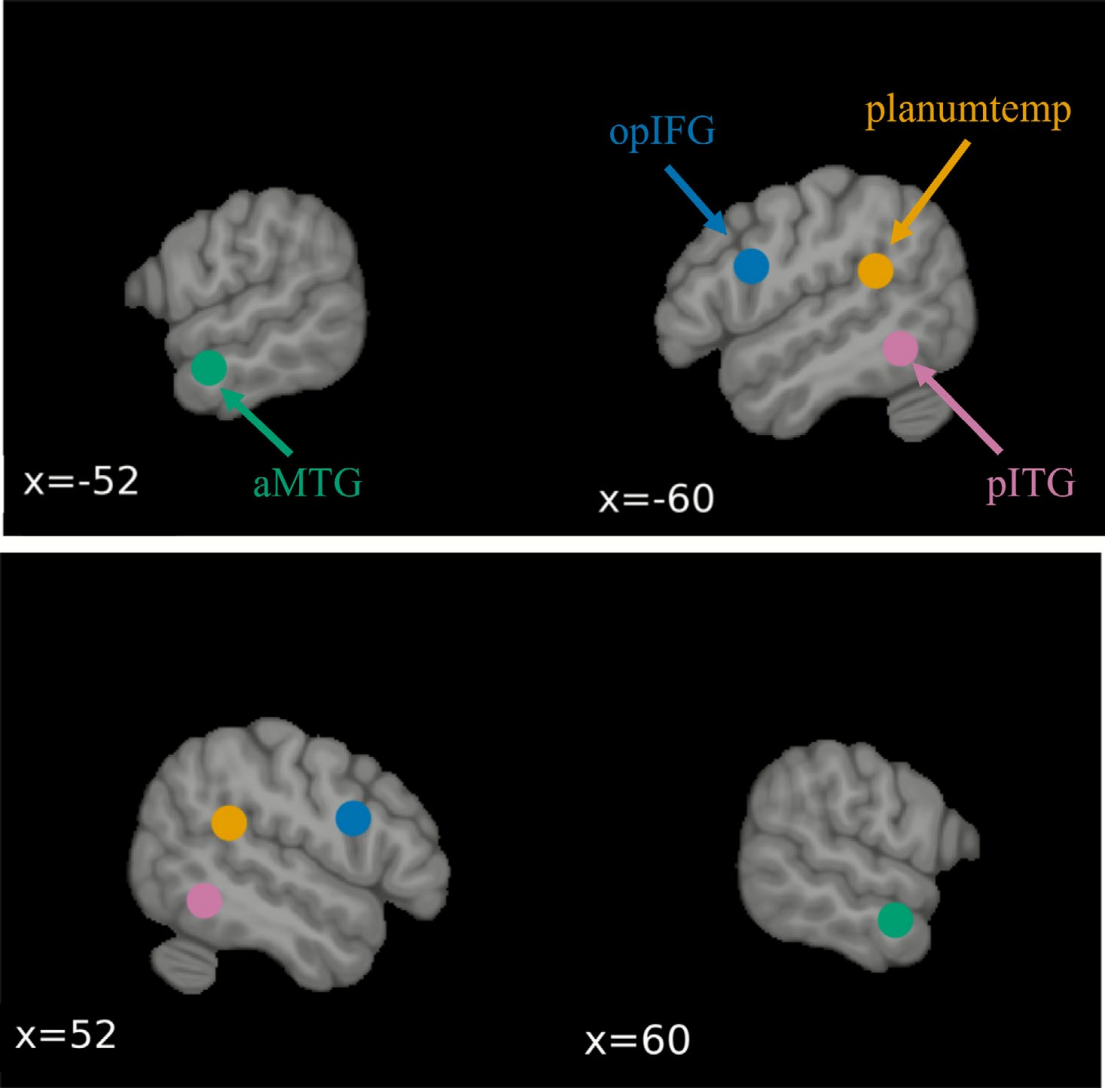
Seed regions used for the seed‐to‐voxel analyses. The seed regions anchored into core regions of the language network, defined from the work of Battistella et al. (2020).

Seed‐to‐voxel analysis of rs‐fMRI was performed in Python using Nilearn (version 0.11.0; https://nilearn.github.io). Seed regions were defined based on the work of Battistella et al. (2020), who identified these regions as central core regions of language processing subsystems. The left opercular part of the inferior frontal gyrus (opIFG), the left planum temporale/parietal operculum (planumtemp), the left anterior middle temporal gyrus (aMTG), and the left posterior inferior temporal gyrus (pITG) have been identified as core regions of the articulatory‐phonological processes, speech perception, semantic processing, and orthographic‐to‐phonological processing, respectively. Specifically, the seed regions were placed in the left opIFG (MNI coordinates: *x* = −50, *y* = 8, *z* = 23), the left planumtemp (MNI coordinates: *x* = −51, *y* = −42, *z* = 21), the left aMTG (MNI coordinates: *x* = −60, *y* = −6, *z* = −18), and the left pITG (MNI coordinates: *x* = −54, *y* = −52, *z* = −10), in addition to their homologs in the right hemisphere (same coordinates but with positive *x* value), for a total of eight seed regions (Figure 1) (Battistella et al. 2020). Each seed region was represented by an 8 mm radius sphere centered on each selected coordinate system.

We extracted the mean BOLD time series from each seed region for each participant and for each of the four HCP resting‐state runs using unsmoothed data, in order to preserve the local specificity of the seed signal (Ely et al. 2016). We then extracted voxel‐wise BOLD time series from the smoothed, whole‐brain preprocessed images (Ely et al. 2016). We computed Pearson correlation coefficients between the seed time series and each brain voxel time series, producing seed‐to‐voxel FC maps for each run and seed. These correlation maps were converted into z‐score maps using Fisher's r‐to‐z transformation, resulting in four z‐maps per seed per participant. These were averaged across four runs to produce a final Fisher z‐map per seed region.

To reduce the dimensionality of the data, the eight FC maps (one for each seed region) were parcellated according to the Destrieux atlas, which is composed of 74 parcels per hemisphere (Destrieux et al. 2010). For each parcel, we calculated the mean correlation value by averaging the Fisher z‐scores of all the voxels within the parcel. Since regional gray matter (GM) density is known to differ between sexes (Ruigrok et al. 2014), we controlled its effect on FC maps. GM density maps provided by the HCP (FreeSurfer outputs) were parcellated using the same Destrieux atlas applied to the FC maps. A mean GM density value was obtained for each parcel. For each seed‐based FC map, we fitted a Generalized Linear Model (ordinary least squares) implemented in the Python module statsmodels (v0.14.1), with FC and GM density values within each parcel as dependent variable and predictor, respectively. The residuals from this regression were retained as corrected FC values. For each participant, we created an initial matrix of 148 seed‐to‐parcel FC corrected values per seed region. Finally, the eight matrices (one for each seed) were concatenated to form a single matrix per participant with dimensions of 8 seed regions × 148 Destrieux parcels. These matrices were used as the input features for the classification models. The code used in this project is available at: https://github.com/Xanthylajoie/sex‐classification‐language‐fc.

### 2.4 Classification Analysis

A ML approach was used to assess how accurately the participants' sex could be classified based on the FC patterns of their language network. This classification process was applied to the FC matrix of all the combined seeds and then to the matrices for each seed region individually, resulting in nine models. A Linear Support Vector Machine classifier (SVC) was trained to classify the subjects' sex based on their FC matrix, which is the most widely used to answer this type of question in this field (Cervantes et al. 2020; Pereira et al. 2009). In addition, recent studies focusing directly on sex differences have demonstrated that linear models tend to outperform nonlinear classifiers (Al Zoubi et al. 2022; Dhamala et al. 2020).

Each FC value between the seed region and the atlas parcel represents a predictor in our classification model. We randomly allocated 20% of the main sample to a holdout set (*n* = 112), which was used to further explore the predictive power of the discriminant features. The remaining 80% (*n* = 452) were used to train and test the models. Then, the train‐test set was split in an 80/20 ratio; the classifier was trained on 80% of the train‐test set (*n* = 361) and tested on the remaining 20% of the train‐test (*n* = 90). A bootstrap resampling procedure was performed to assess which atlas parcels made stable contributions to the model's performance, and to derive confidence intervals as performance metrics. The procedure consisted of randomly sampling 10,000 times the train‐test set with replacement. Linear SVC with a 20‐fold cross‐validation, stratified to preserve the ratio between sexes for each split, was applied to each of these samples. For each iteration, the model performance was evaluated using a holdout set.

To identify the top 10 most important features from the 10,000 bootstrap iterations, the feature weights (coefficients) from each iteration were first transformed to the original feature scale by multiplying them by the standard deviation of the corresponding features. This transformation ensures that the weights are interpretable in the original units of the features because the Linear SVC operates in a standardized space. The transformed weights were averaged across all bootstrap iterations to obtain a single stable measure of the importance of each feature. Finally, the features were ranked based on their mean transformed weights, and the top 10 features with the highest values were extracted as the most stable contributors to the model performance. A permutation test (*n* = 1000) was conducted to determine the statistical significance of the model (Nichols and Holmes 2002).

**FIGURE 2:**
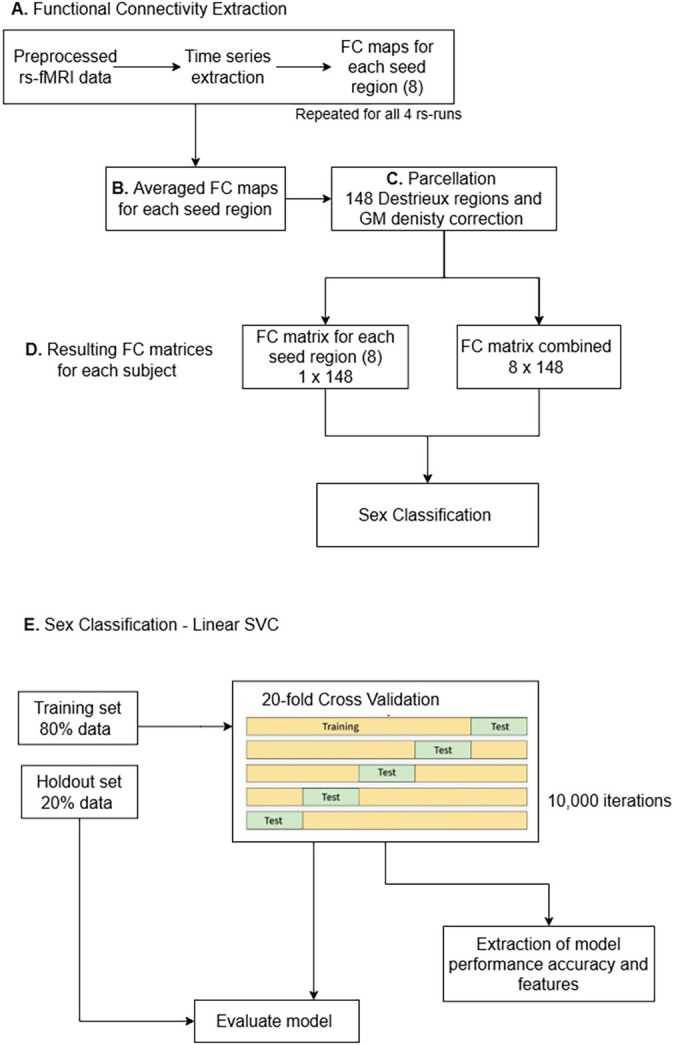
Steps for FC and classification analyses. (A) Time series for each seed region were extracted from preprocessed resting‐state fMRI data. This step was repeated for each rs‐run. (B) The FC maps for each seed region and each run (4) were averaged, resulting in a FC map for each seed region. (C) Each FC map was parcellated according to the Destrieux atlas. (D) Each subject had 8 individual FC matrices, one per seed region, and a FC matrix created by combining the 8 individual matrices to obtain an 8 × 148 FC matrix grouping all seed regions. (E) The classification analysis was separately computed for each individual FC matrix as well as the combined FC matrix to classify sex based on FC. FC, functional connectivity; Linear SVC, linear support vector classifier; rs‐fMRI, resting‐state functional magnetic resonance image.

To further explore the classification results, independent *t*‐tests were conducted to compare the FC values of the top 10 predictors between men and women. This analysis provides an additional descriptive context that the classifier alone does not offer, such as the relative strength of the FC differences between sexes. As a supplementary analysis, we independently selected each discriminant pair identified by the model (Table 4) and evaluated whether the FC pattern between each pair could distinguish between men and women in the holdout sample. An outline of the workflow is shown in Figure 2. All code used in this project can be found at https://github.com/Xanthylajoie/sex‐classification‐language‐fc.

## 3 Results

### 3.1 Sex Classification Results: Accuracy

The linear SVC model based on the eight seeds combined achieved the highest classification accuracy and was able to successfully classify men and women with an average performance accuracy of 91.3% ± 0.03% (95% CI [85.1, 97.6]). The average sensitivity and specificity of the model are 91.2% ± 0.04% and 91.9% ± 0.04% respectively. A permutation test revealed that accuracy was significantly higher than chance (*p* < 0.001). The model had an overall AUC of 0.91 ± 0.03 (95% CI [0.85, 0.98]). When evaluated on the holdout set, the model reached an average accuracy of 78.1% ± 0.03% (95% CI [72.9, 83.3]). The average sensitivity and specificity are 73.6% ± 0.03% and 83.9% ± 0.03%, respectively. The AUC on the holdout set was 0.78 ± 0.03 (95% CI [0.73, 0.84]).

The classification models based on each seed individually successfully classified men and women in the test set with accuracies ranging from 77% to 84%. For each seed, we also evaluated the models' performances on the holdout set, leading to accuracies ranging from 68% to 74.8% (see Table 2). The FC patterns anchored to the left and right opIFG networks and the left pITG network achieved higher classification accuracies (84.0%, 81.4%, and 81.4%, respectively) when tested on the test set, in comparison with the holdout set (73.8%, 72.5%, and 69.4%, respectively).

**TABLE 2:** Classification accuracies of the model when computed for different seed regions on the test set and holdout set.

Seed	Test accuracy (%) ± SD	CI (%)	Sensitivity (%) ± SD	Specificity (%) ± SD	Holdout accuracy (%) ± SD	CI (%)	Sensitivity (%) ± SD	Specificity (%) ± SD
All seeds combined	91.3 ± 0.03	[85.1, 97.6]	91.2 ± 0.04	91.9 ± 0.04	78.1 ± 0.03	[72.9, 83.3]	73.6 ± 0.03	83.9 ± 0.03
opIFG_L	84.0 ± 0.04	[76.1, 91.9]	82.6 ± 0.05	86.5 ± 0.05	73.8 ± 0.03	[68.5, 79.0]	69.4 ± 0.03	79.3 ± 0.04
pITG_L	81.4 ± 0.04	[73.1, 89.8]	80.7 ± 0.05	83.0 ± 0.06	69.4 ± 0.03	[63.9, 74.9]	64.8 ± 0.03	75.9 ± 0.04
opIFG_R	81.4 ± 0.04	[73.2, 89.7]	80.6 ± 0.05	83.0 ± 0.06	72.5 ± 0.03	[67.3, 77.6]	69.0 ± 0.03	76.5 ± 0.04
pITG_R	79.5 ± 0.04	[71.0, 88.0]	78.1 ± 0.05	82.0 ± 0.06	74.8 ± 0.02	[70.1, 79.5]	70.2 ± 0.03	80.8 ± 0.03
Planumtemp_L	79.3 ± 0.04	[70.8, 87.8]	78.1 ± 0.05	81.4 ± 0.06	69.1 ± 0.03	[63.7, 74.4]	65.7 ± 0.03	73.1 ± 0.04
aMTG_L	79.0 ± 0.04	[70.3, 87.6]	77.8 ± 0.05	81.2 ± 0.06	70.3 ± 0.03	[64.3, 76.4]	65.9 ± 0.03	76.4 ± 0.05
aMTG_R	78.9 ± 0.04	[70.3, 87.5]	77.6 ± 0.05	81.1 ± 0.06	68.9 ± 0.03	[63.3, 74.4]	64.0 ± 0.03	76.0 ± 0.04
Planumtemp_R	77.1 ± 0.05	[68.3, 85.9]	75.5 ± 0.05	79.9 ± 0.06	68.0 ± 0.03	[61.9, 74.1]	63.8 ± 0.03	73.7 ± 0.04

*Note:* aMTG_L = left anterior middle temporal gyrus, aMTG_R = right anterior middle temporal gyrus, opIFG_L = left opercular part of the inferior frontal gyrus, opIFG_R = right opercular part of the inferior frontal gyrus, pITG_L = left posterior inferior temporal gyrus, pITG_R = right posterior inferior temporal gyrus, planumtemp_L = left planum temporale, planumtemp_R = right planum temporale.

### 3.2 Differences in the Functional Connectivity Values of the 10 Best Predictors From the Train Test Set

In this section, we present the best predictors for the model trained on all eight combined seeds, which is the model that achieves the highest classification accuracy when tested on both the training test and holdout sets. The 10 best predictors for the models trained on individual seeds are reported in Tables S1–S8.

**FIGURE 3:**
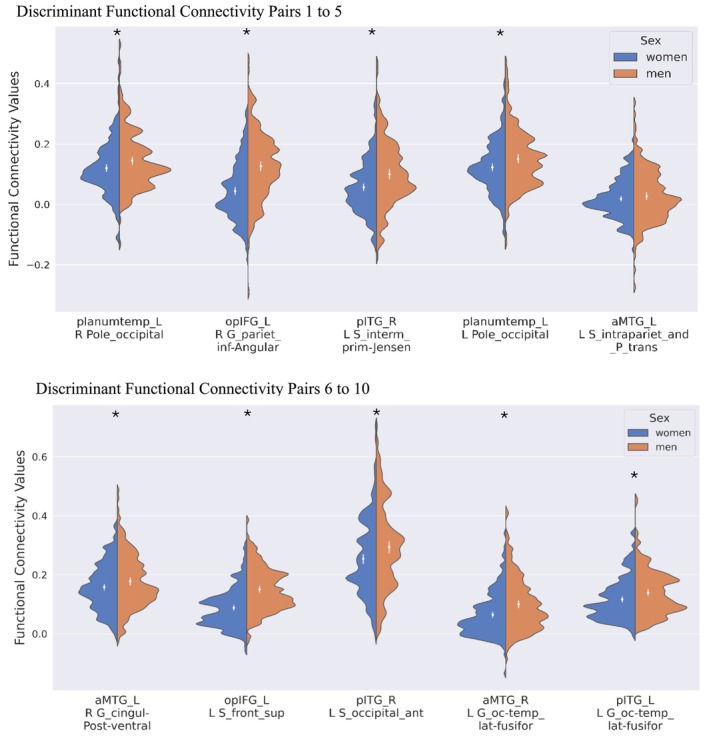
Functional connectivity values for men and women for the top 10 discriminant region pairs listed in Table 3. *Note:* The functional connectivity values (no units) for men and women within each pair are shown vertically. The mean values within each seed‐atlas parcel pairs are indicated by a white circle. aMTG_L = left anterior middle temporal gyrus, opIFG_L = left opercular part of the inferior frontal gyrus, planumtemp_L = left planum temporale, L S_front_sup = left superior frontal sulcus, R G_pariet_inf‐Angular = right angular gyrus, L G_cingul‐Post‐ventral = left posterior‐ventral part of the cingulate gyrus (vPCC), L G_and_S_transv_frontopol = left transverse frontopolar gyri and sulci, L S_intrapariet_and_P_trans = left intraparietal sulcus, R G_cingul‐Post‐ventral = right posterior‐ventral part of the cingulate gyrus (vPCC), R S_front_inf = right superior frontal sulcus, L Pole_occipital = left occipital pole, L G_and_S_cingul‐Ant = left anterior part of the cingulate gyrus and sulcus (ACC), R S_orbital_lateral = right lateral orbital sulcus. *Pairs that differ significantly between men and women.

The 10 seed‐to‐atlas parcel pairs with the highest predictive power in the 8 seeds combined model included the pairs between the left planum temporale and the left and right occipital poles, between the left opIFG and the right angular gyrus, the left superior frontal sulcus, between the left aMTG and the left intraparietal sulcus and right posterior‐ventral part of the cingulate gyrus (vPCC), between the right aMTG and the left lateral occipitotemporal gyrus, between the right pITG and the left sulcus intermedius primus (of Jensen) and the left anterior occipital sulcus. The 10 best predictors (seed‐to‐atlas parcel pairs) and mean *z*‐score values for men and women are reported in Table 3 and visually represented in Figure 3.

**TABLE 3:** Mean functional connectivity values for men and women in the top 10 predictive seed to parcel pairs identified by the model with all seeds combined in the train test sample.

Seed region—Destrieux atlas parcel	Men (mean ± SD)	Women (mean ± SD)	*t*	*p*
Planumtemp_L—Right occipital pole	0.15 ± 0.10	0.12 ± 0.19	2.88	0.004*
opIFG_L—Right angular gyrus	0.13 ± 0.11	0.04 ± 0.09	8.36	< 0.001*
pITG_R Left Sulcus intermedius primus (of Jensen)	0.10 ± 0.11	0.06 ± 0.09	4.64	< 0.001*
Planumtemp_L—Left occipital pole	0.15 ± 0.10	0.12 ± 0.08	3.26	0.001*
aMTG_L—Left intraparietal sulcus	0.03 ± 0.09	0.02 ± 0.06	1.22	0.22
aMTG_L—Right posterior‐ventral part of the cingulate gyrus (vPCC)	0.18 ± 0.09	0.16 ± 0.08	2.48	0.01*
opIFG_L—Left superior frontal sulcus	0.15 ± 0.07	0.09 ± 0.07	9.39	< 0.001*
pITG_R—Left Anterior occipital sulcus	0.29 ± 0.14	0.25 ± 0.13	3.12	0.002*
aMTG_R—Left Lateral occipito‐temporal gyrus	0.10 ± 0.09	0.06 ± 0.07	4.73	< 0.001*
pITG_L—Left Lateral occipito‐temporal gyrus	0.14 ± 0.08	0.12 ± 0.07	3.28	0.001*

*Note:* aMTG_L = left anterior middle temporal gyrus, aMTG_R = right anterior middle temporal gyrus, opIFG_L = left opercular part of the inferior frontal gyrus, planumtemp_L = left planum temporale, pITG_L = left posterior inferior temporal gyrus, pITG_R = right posterior inferior temporal gyrus.

* Pairs that differ significantly between men and women.

The *t*‐test supplementary analysis comparing the mean *z*‐score values between men and women of the 10 best predictors revealed significant differences in the strength of connectivity in nine out of 10 seed‐to‐atlas parcel pairs (identified with an asterisk in Table 3 and Figure 3). In all the significant pairs, the connectivity values were higher in men than in women. This analysis was performed for further characterization; although a predictor might be highly discriminant in the classification model, this does not necessarily mean that the FC values within these regions are significantly different between men and women. This can be seen between the connectivity of the left aMTG and the left intraparietal sulcus, which shows no significant differences for sex in the strength of connectivity but still remains a discriminant pair identified by the classification model. In addition, the violin plot (Figure 3) showed a large degree of overlap between the FC values of men and women across all seed‐to‐parcel pairs.

To illustrate how the networks anchored to each seed region are organized in men and women, the FC values between each seed and parcels are visually represented on a brain template in Figures S1–S8.

### 3.3 Classification Based on Each Discriminant Pair

In this section, we present the classification accuracy of each discriminant pair when tested independent of the holdout set. The classification accuracies ranged from 39% to 65%, with the highest being the pattern between the left planum temporale and the right occipital pole and the lowest being the FC pattern between the left aMTG and the left intraparietal sulcus. The results are summarized in Table 4.

**TABLE 4:** Predictive Power of each discriminant feature when tested on the holdout set.

Seed region—Destrieux atlas parcel	% Accuracy	% Sensitivity	% Specificity
Planumtemp_L—Right occipital pole	65.2	63.6	66.7
opIFG_L—Right angular gyrus	52.2	50.0	53.3
pITG_R Left Sulcus intermedius primus (of Jensen)	52.1	50.0	53.8
Planumtemp_L—Left occipital pole	52.2	50.0	53.8
aMTG_L—Left intraparietal sulcus	39.1	28.6	43.8
aMTG_L—Right posterior‐ventral part of the cingulate gyrus (vPCC)	43.5	33.3	47.1
opIFG_L—Left superior frontal sulcus	60.9	66.7	58.8
pITG_R—Left Anterior occipital sulcus	65.2	63.6	66.7
aMTG_R—Left Lateral occipito‐temporal gyrus	47.8	44.4	50.0
pITG_L—left Lateral occipito‐temporal gyrus	52.2	50.0	53.3

## 4 Discussion

The results of this study showed that, when combining connectivity data from all seed regions, the classifier achieved a high accuracy of 91.3% on the train‐test set. This performance surpasses that of models based on individual seed regions, highlighting the value of integrating connectivity data across language subnetworks to improve classification. The best predictors for the model based on all eight combined seeds were mainly anchored to the left hemisphere, more specifically, to the left planum temporale, left opIFG, left aMTG, and right pITG. While there was considerable overlap in connectivity patterns between men and women, significant sex differences were observed in connectivity strength across all discriminant pairs, with men displaying higher FC values than women. These results demonstrate that the FC characteristics of language subnetworks contain key information to distinguish between the sexes. Considering that language‐related disorders, such as speech and language impairments, have been associated with altered FC patterns and that these disorders often differ by sex, it is important to understand whether sex has an impact on FC patterns within the language network.

To the best of our knowledge, this study is the first to classify sex based on rs‐FC patterns anchored to core regions of the language network, using a ML approach. Recent studies also using the HCP dataset have classified sex based on whole brain rs‐FC data and reported test accuracies of up to 86.6% for whole‐brain FC (Zhang et al. 2018) and 75.1% for parcel‐specific FC (Weis et al. 2020). Another recent study tested various classification models and atlases, achieving accuracy over 80% using whole‐brain rs‐FC patterns (Al Zoubi et al. 2022). In our study, we achieved a similar accuracy range when analyzing the seed regions separately in the classification model. However, when all the seeds were combined into a single model, the classification accuracy reached 91.3% on the train‐test set, surpassing previously published results. Our findings underscore the importance of examining the language network as a whole, incorporating all the seed regions critical for different language subnetworks, rather than focusing on isolated seed regions. Using multiple seed regions allows the classifier to capture a more comprehensive overview of the information from multiple subnetworks of the language network. In addition, combining multiple seed regions can help mitigate the impact of noise or variability in individual regions, leading to a more accurate classification model.

Previous studies have provided evidence that the rs‐FC patterns of the language network could be key contributors to sex classification (Al Zoubi et al. 2022; Weis et al. 2020; Zhang et al. 2018). In particular, the left inferior frontal gyrus, bilateral temporal lobes, and poles were highlighted in these studies (Al Zoubi et al. 2022; Weis et al. 2020; Zhang et al. 2018). Consistent with these results, when we computed the classification model based on the rs‐FC of each seed region separately, the FC maps anchored to the left and right opIFG and left pITG had the highest accuracies. In addition, we used the bootstrap procedure to extract the most reliable discriminant seed‐region‐atlas‐parcel pairs, which were the most consistently reported across 10,000 iterations within the model combining all eight seeds. The top 10 discriminant FC patterns extracted from the bootstrap procedure were mostly anchored to the left planum temporale, left opIFG, left aMTG, and right pITG seed regions and involved regions directly linked to language processing, as well as other regions involved in broader cognitive networks.

In this study, the top predictive FC pattern was between the left planum temporale and right occipital pole, which showed significant sex differences in the strength of connectivity. To date, functional studies focusing on the planum temporale are scarce and have yielded mixed results. While some studies have demonstrated that the activation patterns are asymmetrical between men and women in this area (Baxter et al. 2003; Kansaku et al. 2000; Phillips et al. 2001), others have not found any differences (Halari et al. 2006; Sommer et al. 2008). To the best of our knowledge, sex differences in the FC patterns in this region have not been investigated.

The left opIFG seed region, which is renowned for its critical role in speech production (Battistella et al. 2020; Keller et al. 2009), shows discriminant FC with several brain areas linked to language processing, specifically with regions along the dorsal pathway. Notably, this seed demonstrates strong connectivity with the left superior frontal sulcus, which plays a role in executive function, and the right angular gyrus, a region involved in semantic processing and reading comprehension (du Boisgueheneuc et al. 2006; Kuhnke et al. 2023). These two pairs were among the 10 most discriminative features contributing to sex classification, with men showing a significantly stronger FC than women. Together, these findings suggest that the left opIFG is integrated with regions supporting language, and that sex may influence how these integrative pathways are functionally organized.

The left aMTG seed region, which is involved in semantic processing (Battistella et al. 2020), showed discriminant connectivity with the right vPCC, a key region of the default mode network that is also implicated in semantic processing and integration of linguistic information (Muller and Meyer 2014). The left aMTG also exhibited discriminant FC with the left intraparietal sulcus, a region proposed to function as a semantic hub in the brain (Zhao et al. 2017). These results support the hypothesis that FC patterns within the subnetwork of speech production anchored to the left opIFG and the semantic subnetwork anchored to the left aMTG contain critical information capable of predicting sex. Previous classification studies reported parcels with the highest classification accuracies, but failed to report the specific connections of these parcels. In this sense, our study provides novel information concerning the seed‐to‐parcel pairs of the semantic network and speech production subnetworks, which are the most discriminant for sex classification.

Although ML models identify discriminative features that allow classification, these features are not guaranteed to differ significantly between groups. In our study, nine out of ten discriminant pairs identified by the model combining all eight seed regions were significant for sex differences in terms of FC strength. These differences were observed despite a general overlapping pattern of FC between men and women (Figure 3 and Figures S1–S8). This overlap is not unique to the language network; evidence from other studies has shown that FC patterns between sexes often exhibit large overlaps across various brain networks (Weis et al. 2020; Zhang et al. 2016; Zhang et al. 2018). However, the analysis of the strength of FC within the discriminant features between men and women revealed that men tended to exhibit overall higher FC values than women across all 10 top predictive pairs identified. These results are consistent with those of previous studies that used the same HCP S1200 dataset and consistently reported higher FC values in men across various cognitive networks (Dhamala et al. 2020; Zhang et al. 2016; Weis et al. 2020). Interestingly, studies using different datasets have reported contrasting results, with some suggesting higher FC values in women (Bluhm et al. 2008; Tomasi and Volkow 2012; Weissman‐Fogel et al. 2010). These discrepancies may arise from variations in sample size, analysis methods, or population characteristics, highlighting the importance of considering dataset‐specific factors when interpreting the results.

Another factor that highlights the importance of investigating the generalizability of findings in classification studies is the classification performance of the holdout sample. Although we achieved an accuracy of 91.3% in predicting sex using our training–test data, the model's performance dropped to 78.1% when evaluated using the holdout set. While this still suggests some generalizability potential, it highlights a notable difference compared to the training–test performance accuracy. Independent verification has not been commonly performed in prior studies; however, similar or lower accuracies have been reported (Weis et al. 2020). Future studies should consider improving the generalizability of ML results by using more diverse sample sets and validating the findings on independent datasets.

Studying the link between the brain and language performance was beyond the scope of the present study. Thus, no conclusions can be drawn regarding whether the observed FC patterns have behavioral relevance. Furthermore, our study focused on biological sex because gender identity data were not available. Future studies should aim to disentangle the complex relationship between sex and gender in the functional organization of the language network.

In conclusion, our findings reveal that the FC connectivity pattern anchored to the core regions of language subnetworks could accurately classify sex, with the highest performance achieved when combining all seed regions. These results emphasize the critical role of sex as a biological variable that shapes the functional organization of a language network. Our findings provide a foundation for exploring, in the future, how these connectivity patterns might contribute to sex‐specific risks and outcomes in conditions like aphasia and other language network disorders.

## Funding

X.L. is funded by a scholarship by the Fonds de recherche du Québec—Nature et technologies (2020‐RS4‐265502—Centre UNIQUE—Union Neurosciences & Artificial Intelligence—Quebec). The study is funded by the Natural Sciences and Engineering Research Council of Canada (NSERC) and the Chaire Courtois en recherche fondamentale III (neuroscience) de l’Université de Montréal.

## Ethics Statement

The project has been approved by the comité d’éthique de la recherche—vieillissement et neuroimagerie (CÉR VN) of the du CIUSSS Centre‐Sud‐de‐l'île‐de‐Montréal (CER VN du CCSMTL) (approbation CER VN 22‐23‐02).

## Conflicts of Interest

The authors declare no conflicts of interest.

## Supporting information


**Figure S1:** Connectome showing the network anchored to the left aMTG in men and women.
**Figure S2:** Connectome showing the network anchored to the right aMTG in men and women.
**Figure S3:** Connectome showing the network anchored to the left opIFG in men and women.
**Figure S4:** Connectome showing the network anchored to the right opIFG in men and women.
**Figure S5:** Connectome showing the network anchored to the left pITG in men and women.
**Figure S6:** Connectome showing the network anchored to the right pITG in men and women.
**Figure S7:** Connectome showing the network anchored to the left planum temporale in men and women.
**Figure S8:** Connectome showing the network anchored to the right planum temporale in men and women.

**Table S1:** Top 10 Discriminant Functional Connectivity Patterns Between Men and Women Anchored to the Left aMTG.
**Table S2:** Top 10 discriminant functional connectivity patterns between men and women anchored to the right aMTG.
**Table S3:** Top 10 discriminant functional connectivity patterns between men and women anchored to the left opIFG.
**Table S4:** Top 10 discriminant functional connectivity patterns between men and women anchored to the right opIFG.
**Table S5:** Top 10 discriminant functional connectivity patterns between men and women anchored to the left pITG.
**Table S6:** Top 10 discriminant functional connectivity patterns between men and women anchored to the right pITG.
**Table S7:** Top 10 discriminant functional connectivity patterns between men and women anchored to the left planumtemp.
**Table S8:** Top 10 discriminant functional connectivity patterns between men and women anchored to the right planumtemp.

## Data Availability

The data that support the findings of this study are openly available in Human Connectome Project at https://www.humanconnectome.org/study/hcp‐young‐adult/document/extensively‐processed‐fmri‐datadocumentation (Van Essen et al. 2012). All code for data analysis associated with the current submission is available at https://github.com/Xanthylajoie/sex‐classification‐language‐fc.
