# Supplementary material for: Sex Classification Based on the Functional Connectivity Patterns of the Language Network: A Resting State fMRI Study
Source: Hum Brain Mapp. 2026 Jan 10;47(1):e70450. doi: 10.1002/hbm.70450 (PMC12790092; doi:10.1002/hbm.70450)

Supplementary Figures

Note: The color bar on the right represents functional connectivity values. There were no negative values therefore the minimum value is 0.

**Figure 1**

*Connectome showing the network anchored to the left aMTG in men and women.*


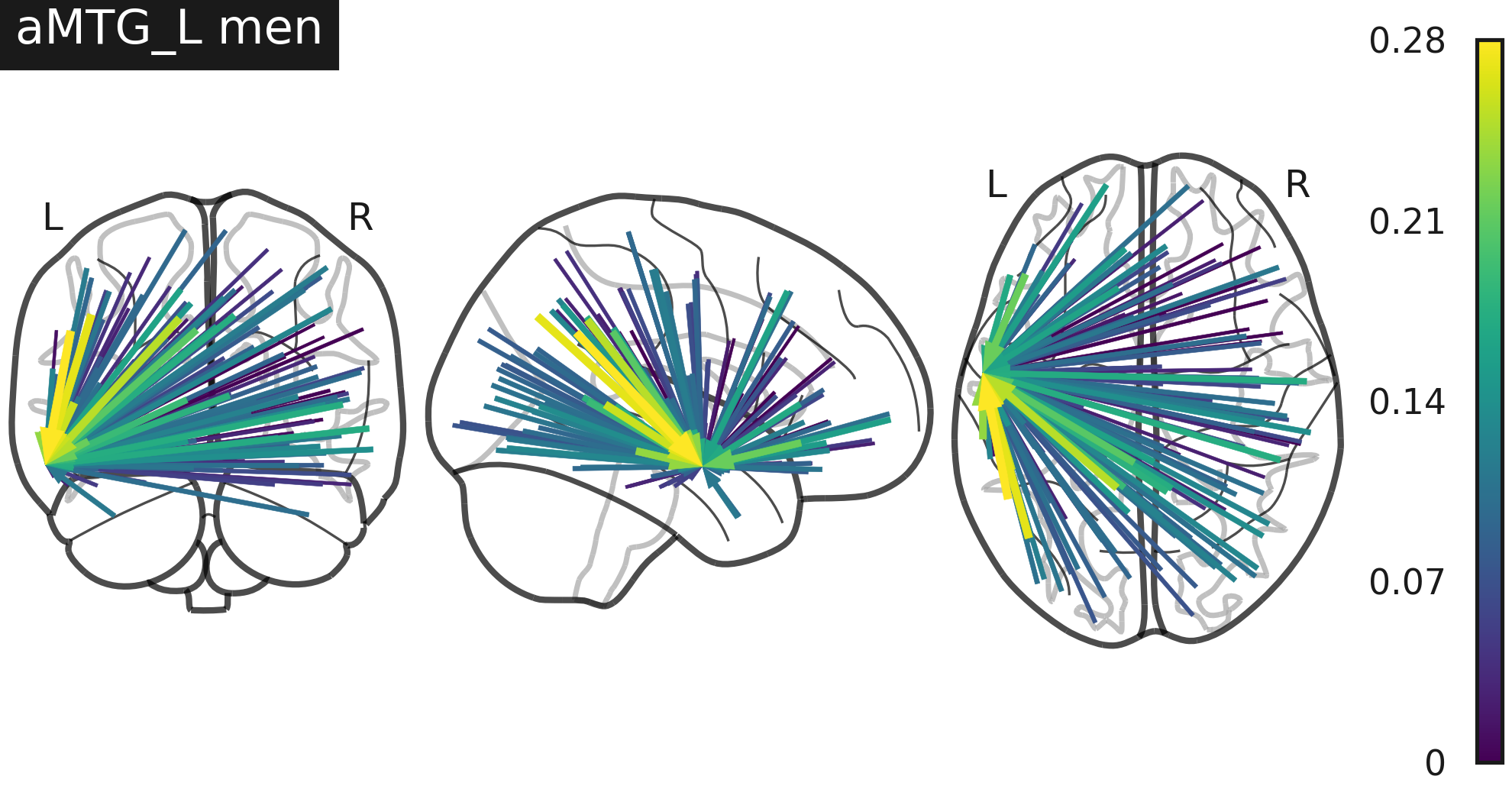

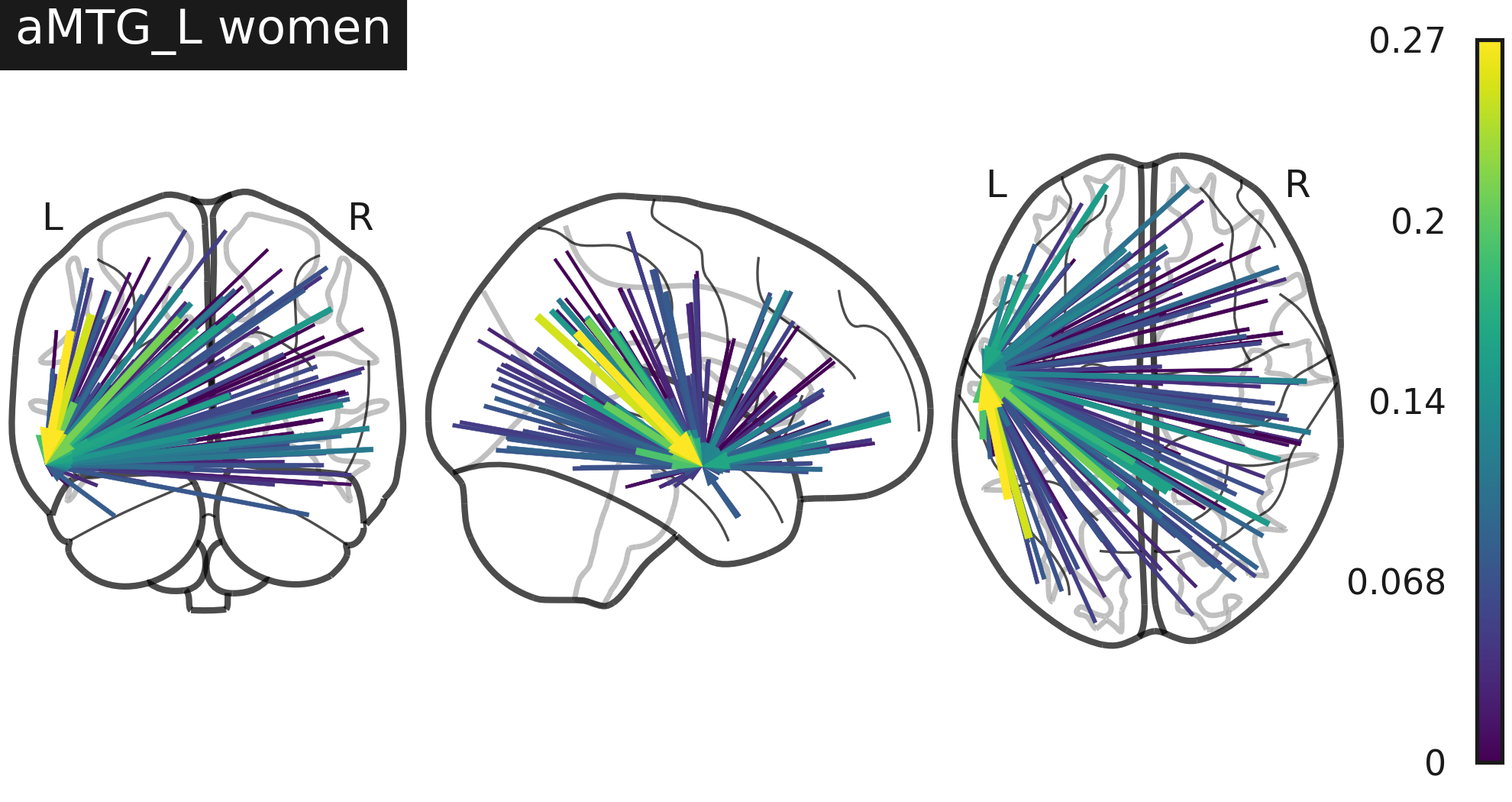


**Figure 2**

*Connectome showing the network anchored to the right aMTG in men and women.*


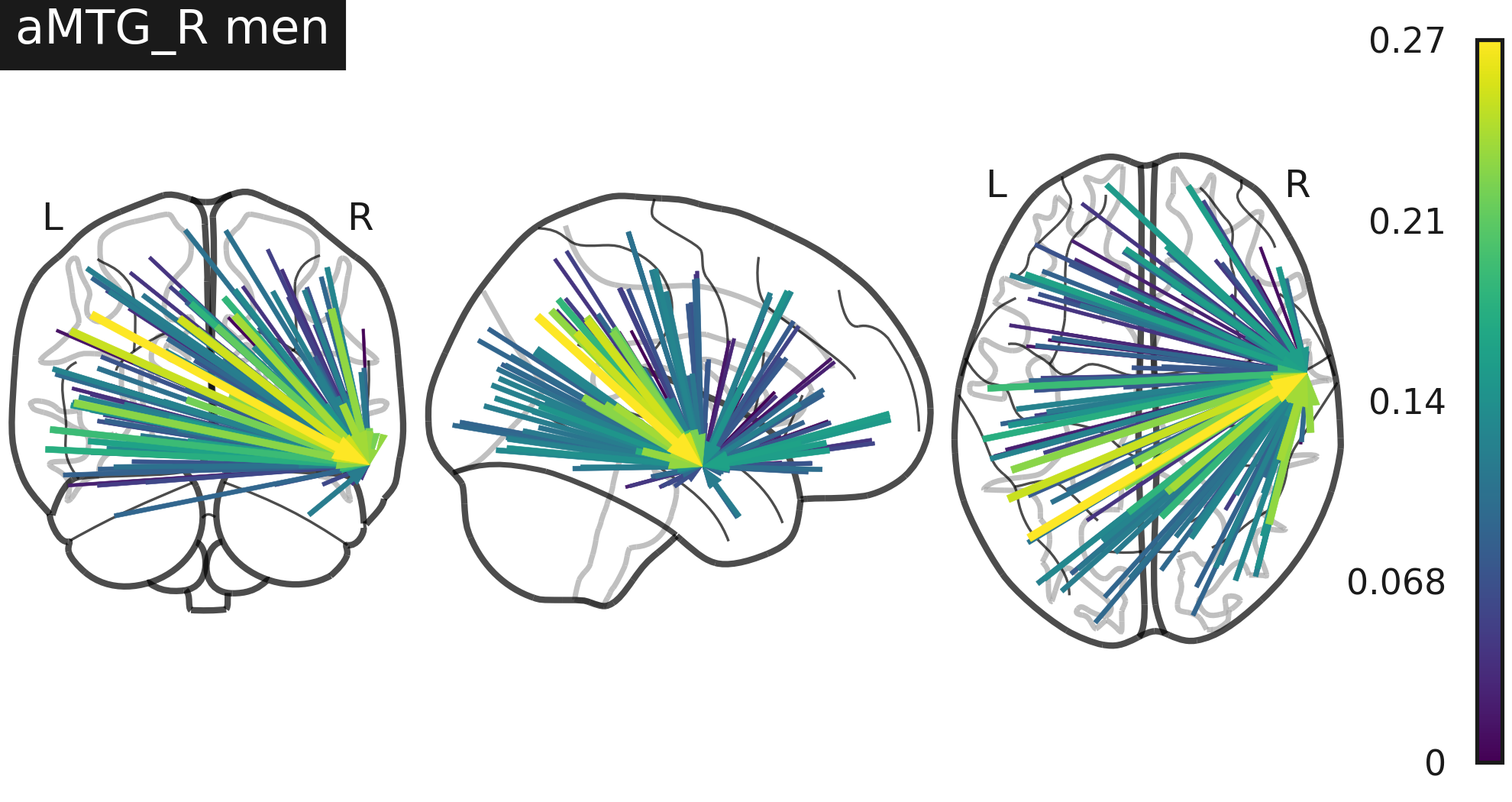


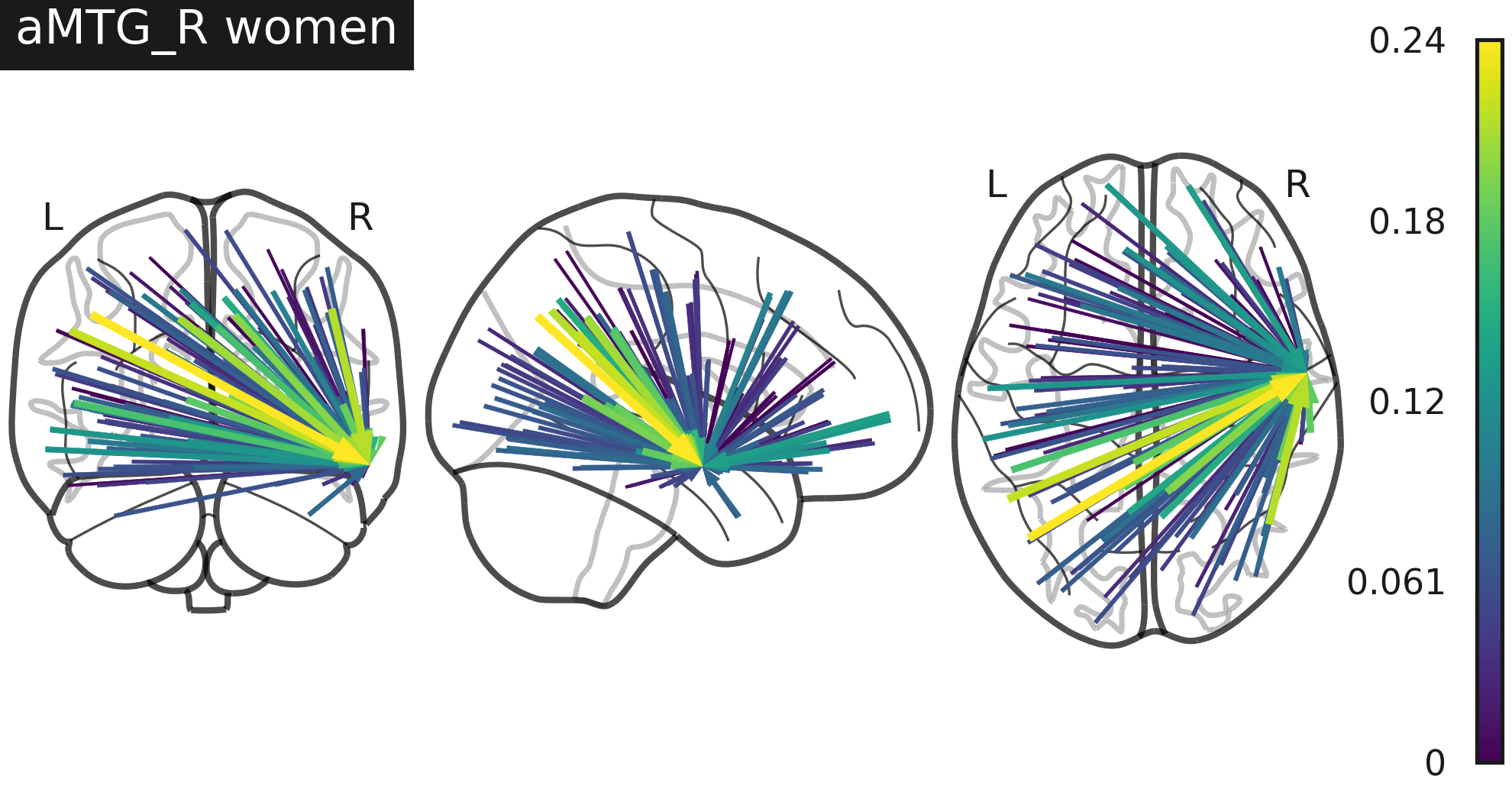


**Figure 3**

*Connectome showing the network anchored to the left opIFG in men and women.*


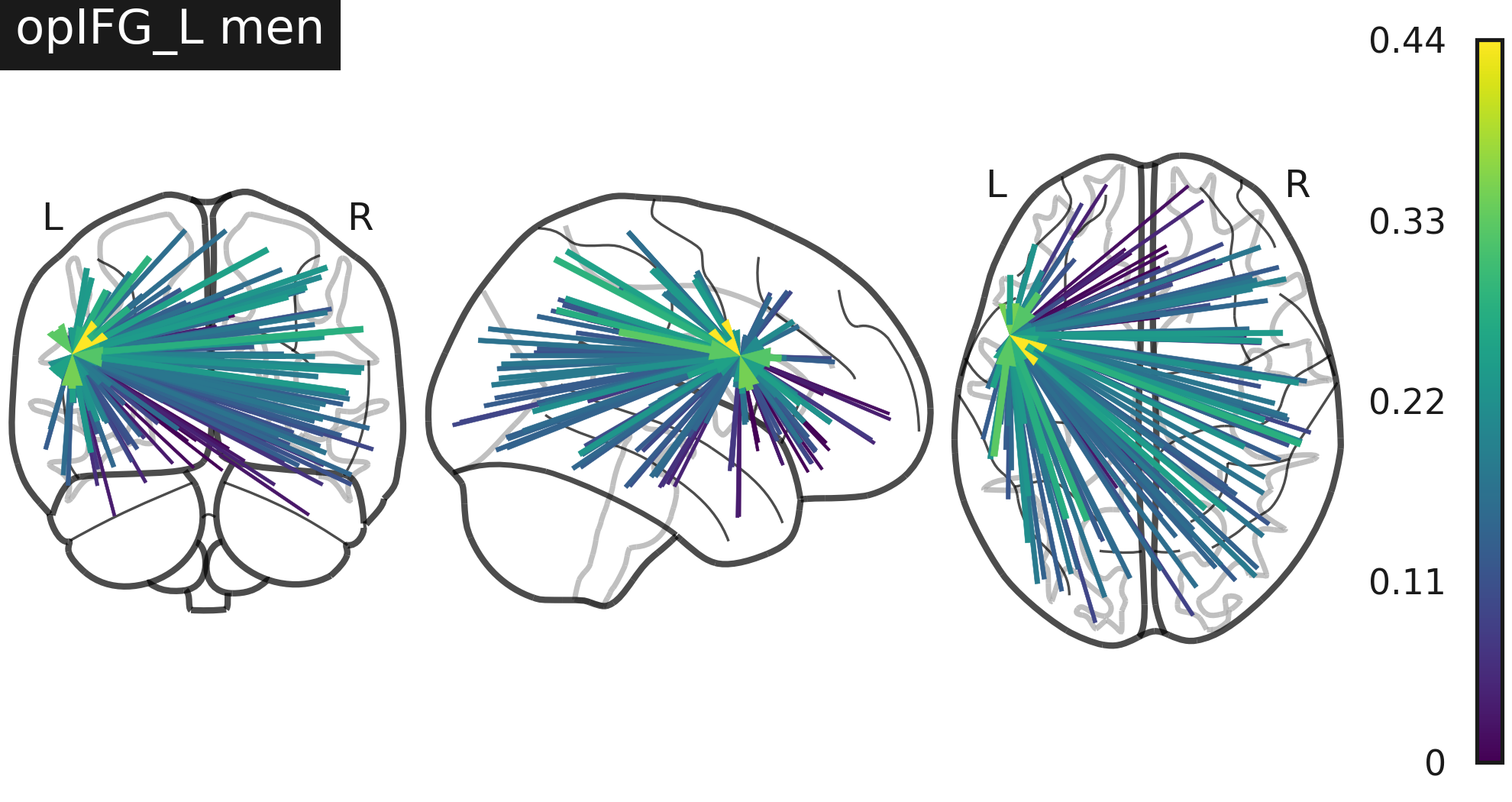


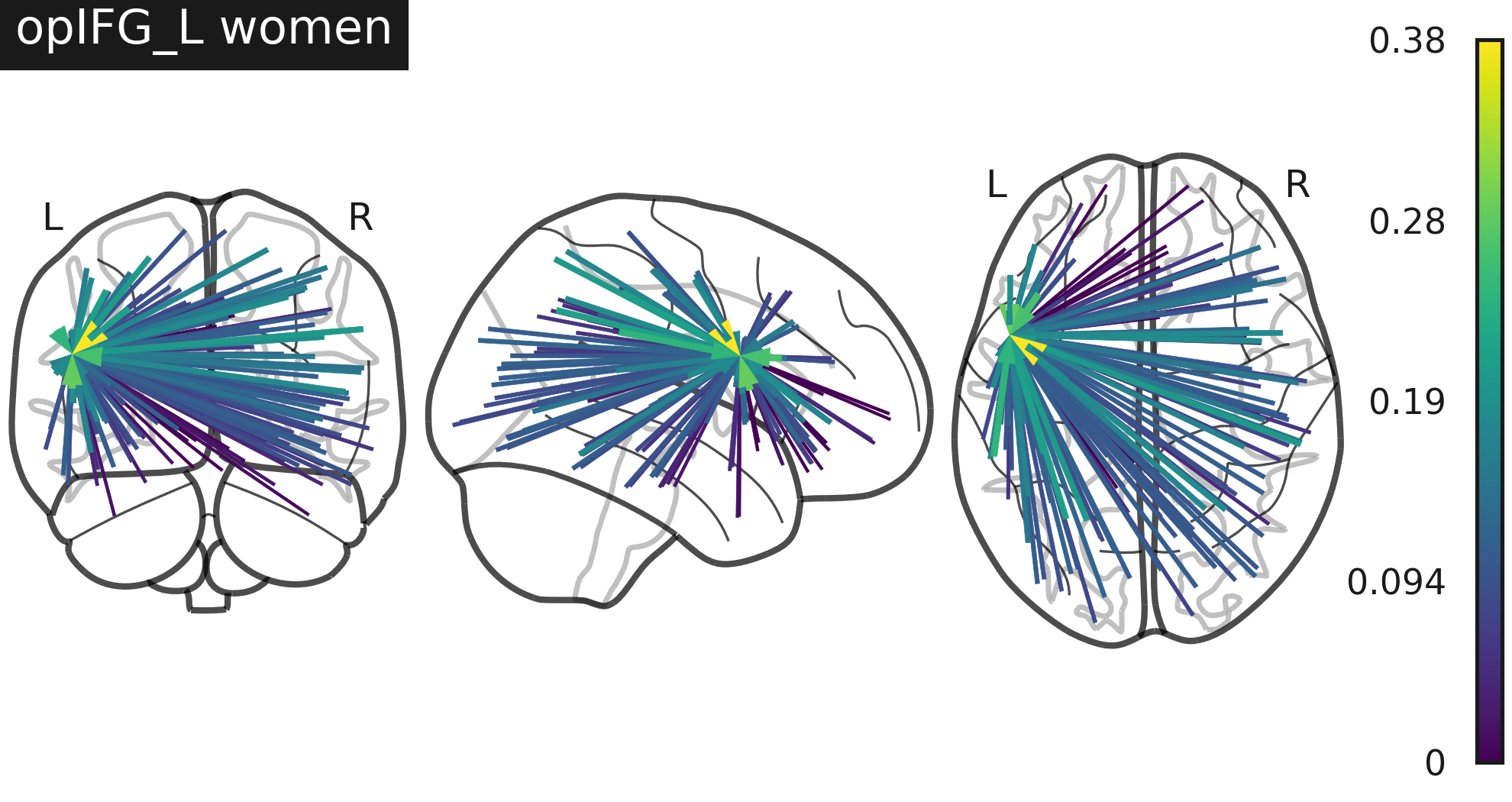


**Figure 4**

*Connectome showing the network anchored to the right opIFG in men and women.*


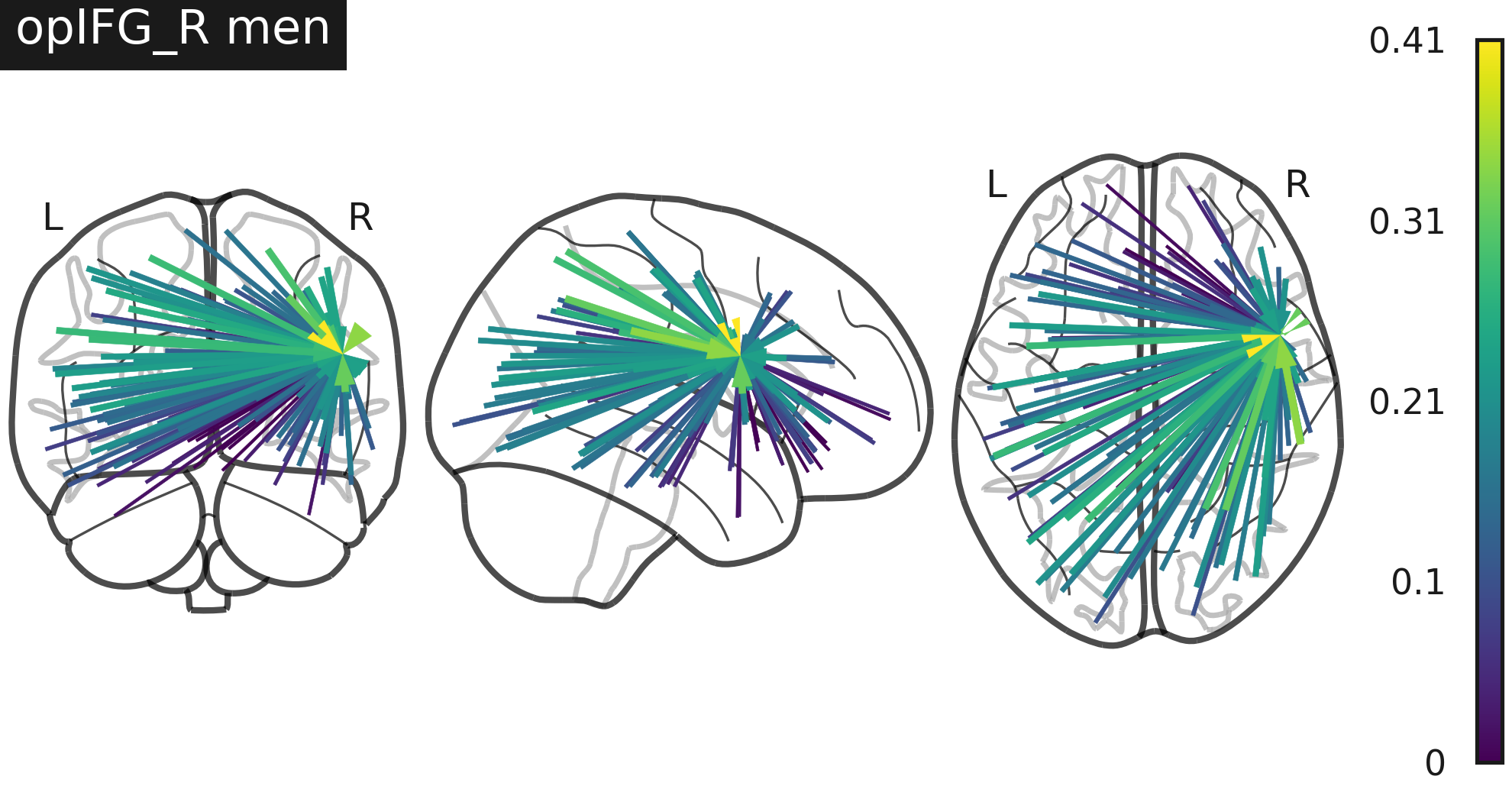


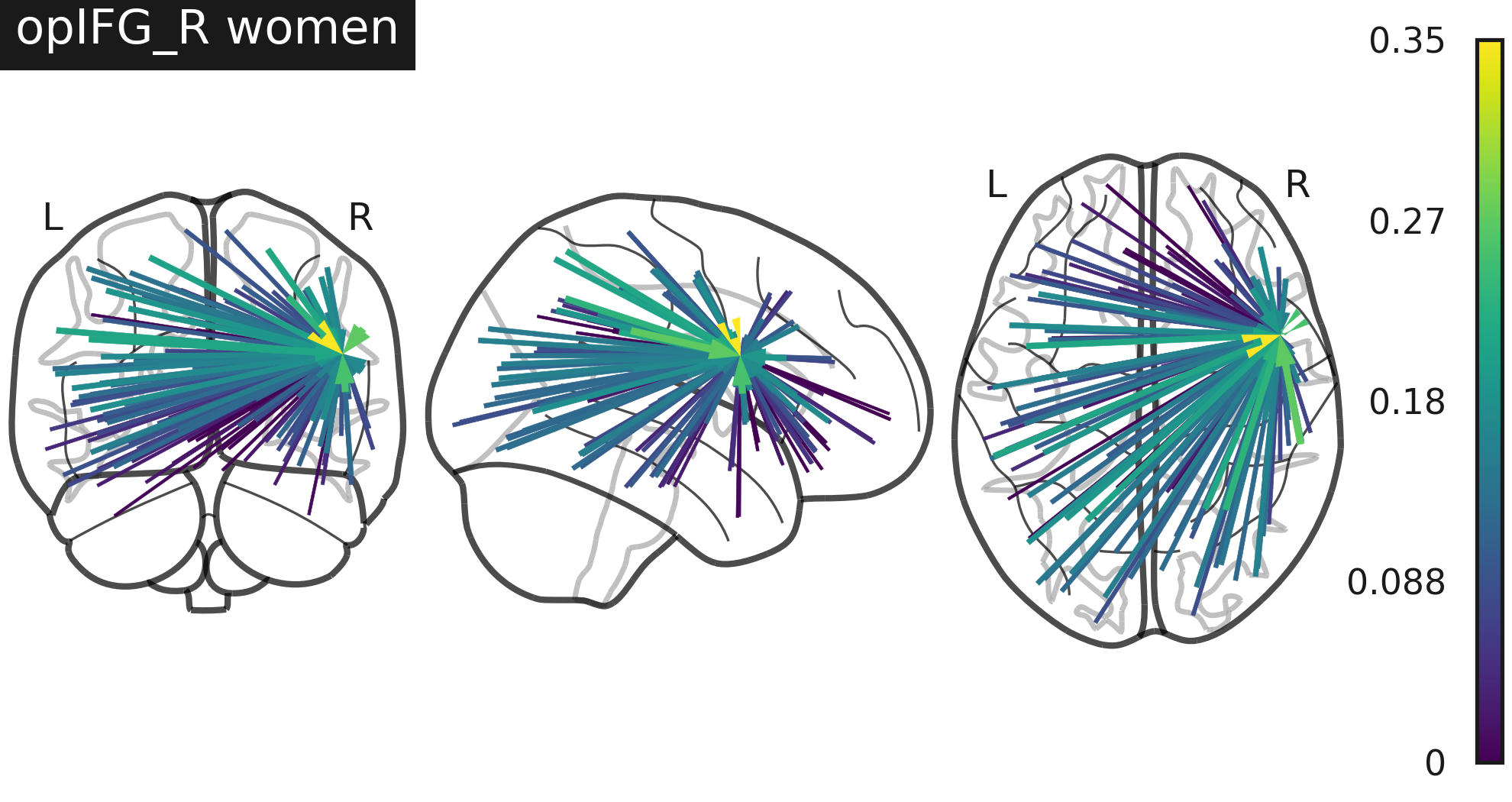


**Figure 5**

*Connectome showing the network anchored to the left pITG in men and women.*


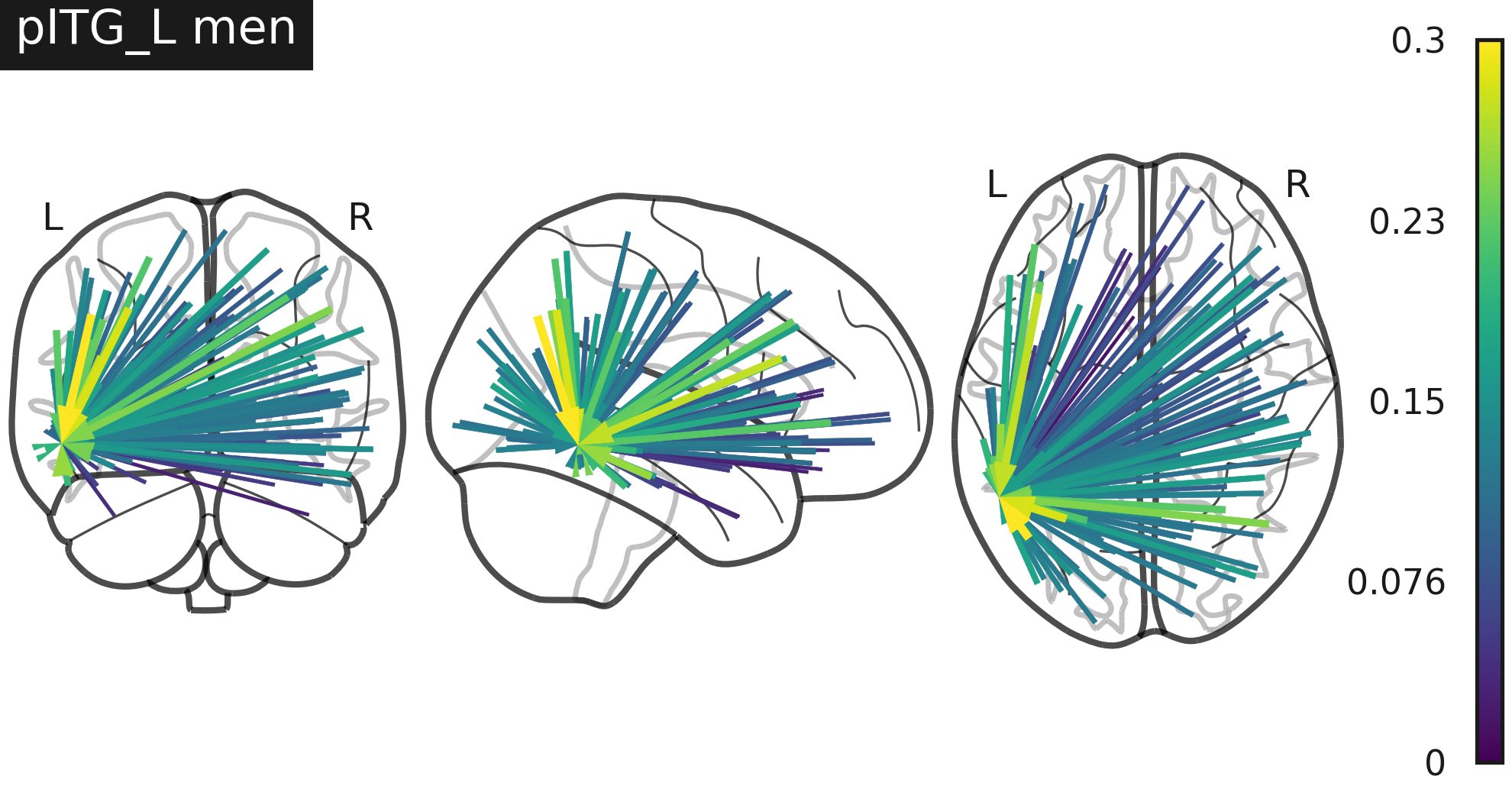


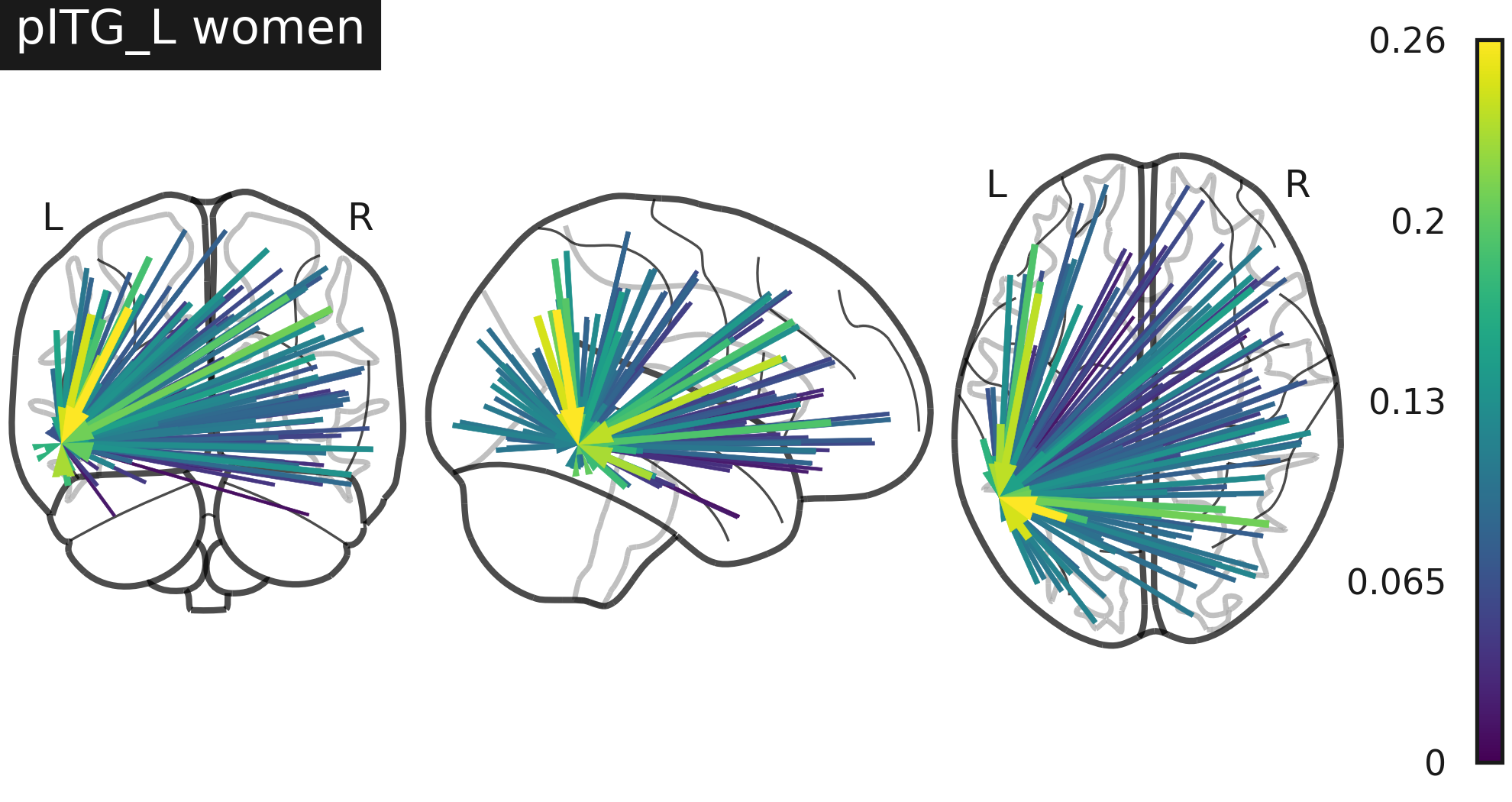


**Figure 6**

*Connectome showing the network anchored to the right pITG in men and women.*


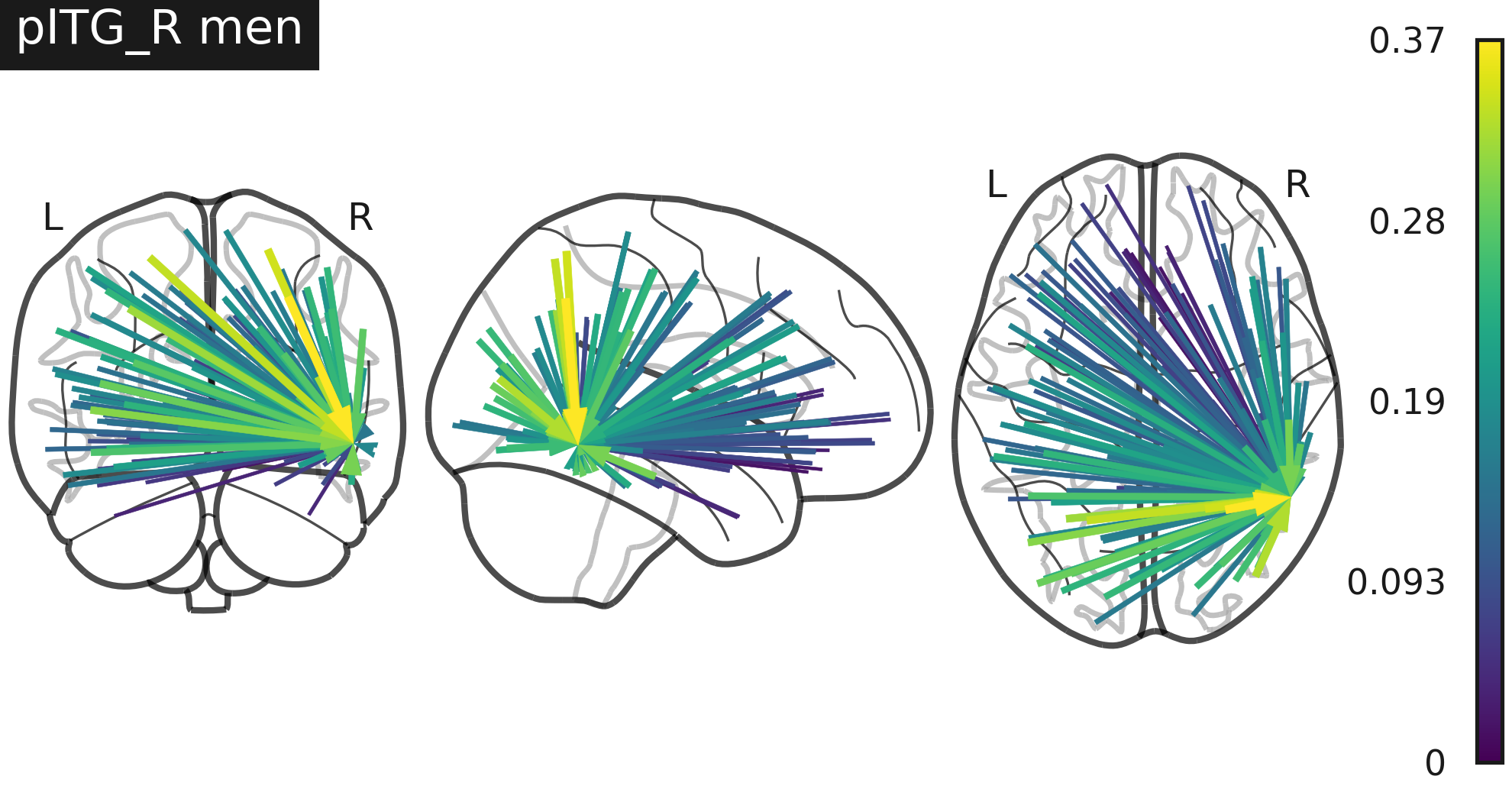


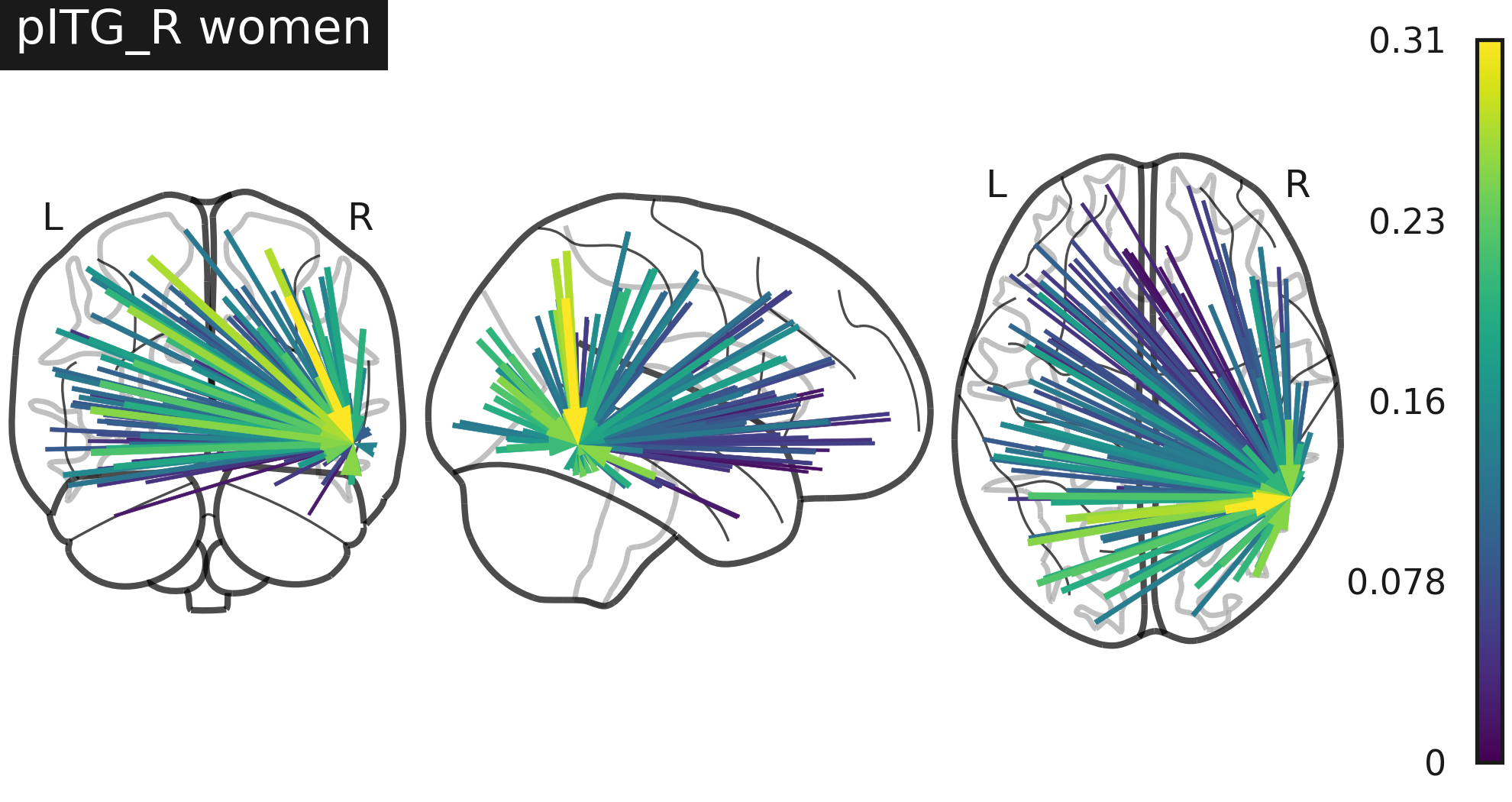


**Figure 7**

*Connectome showing the network anchored to the left planum temporale in men and women.*


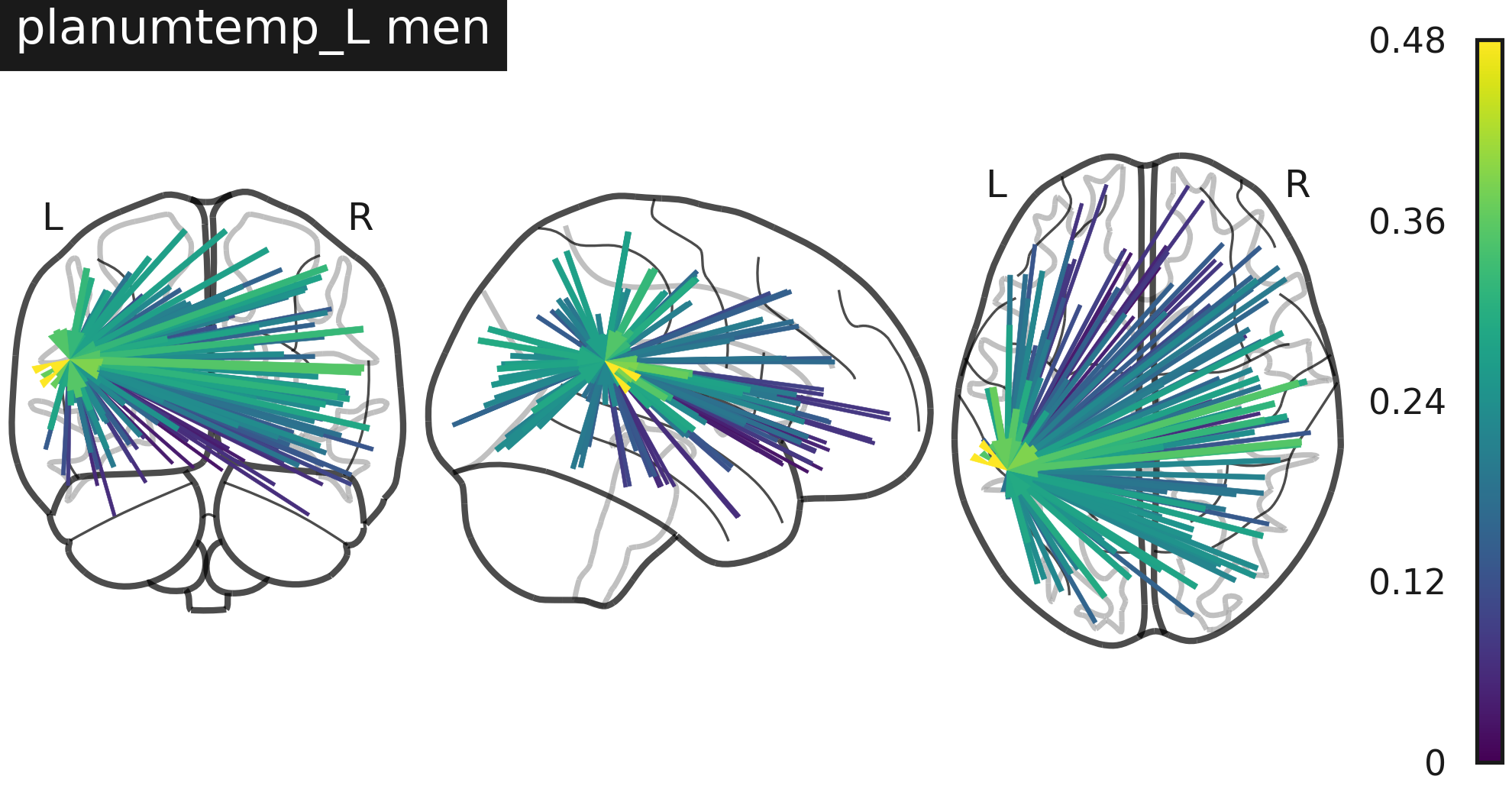


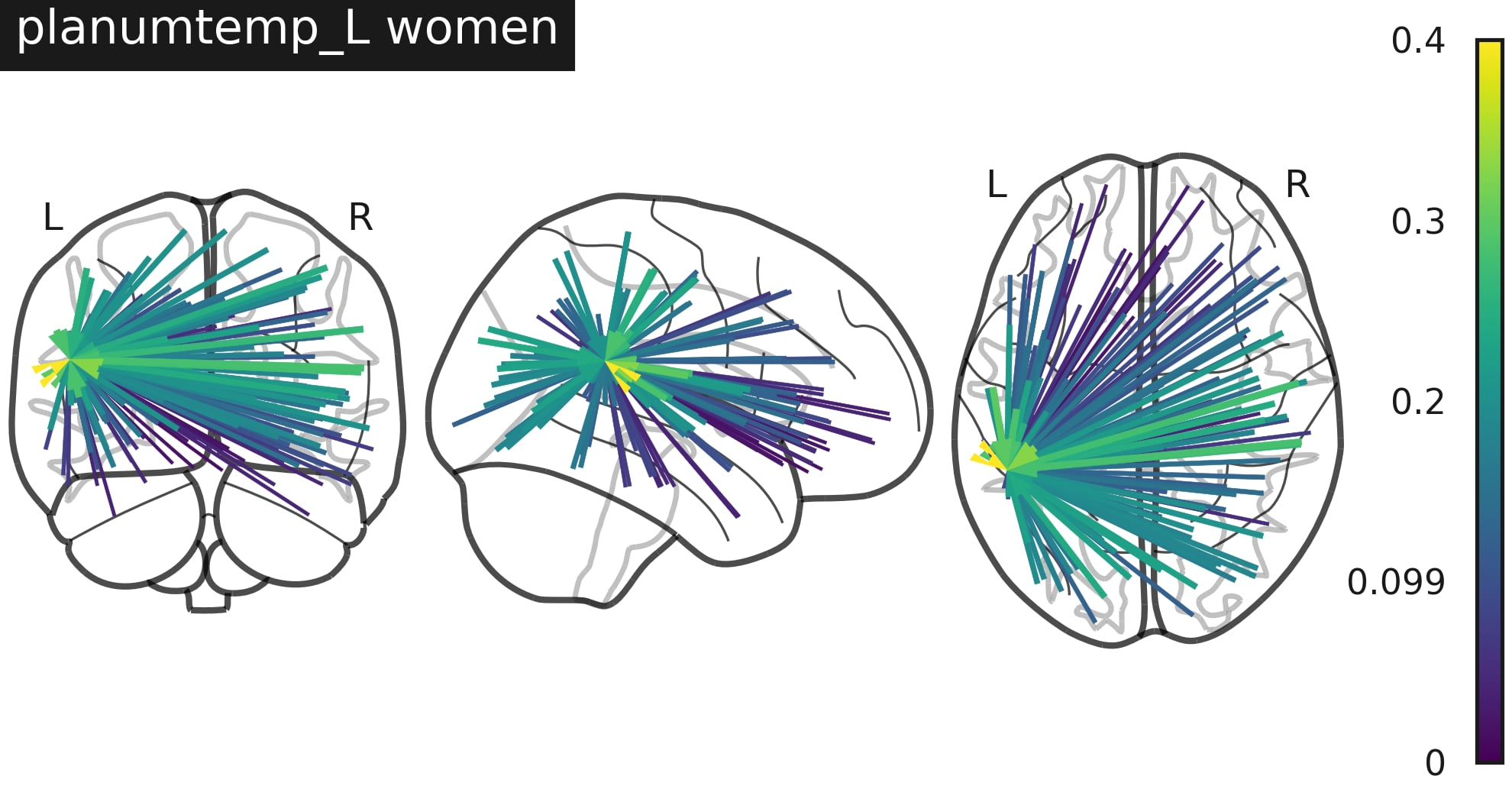


**Figure 8**

*Connectome showing the network anchored to the right planum temporale in men and women.*


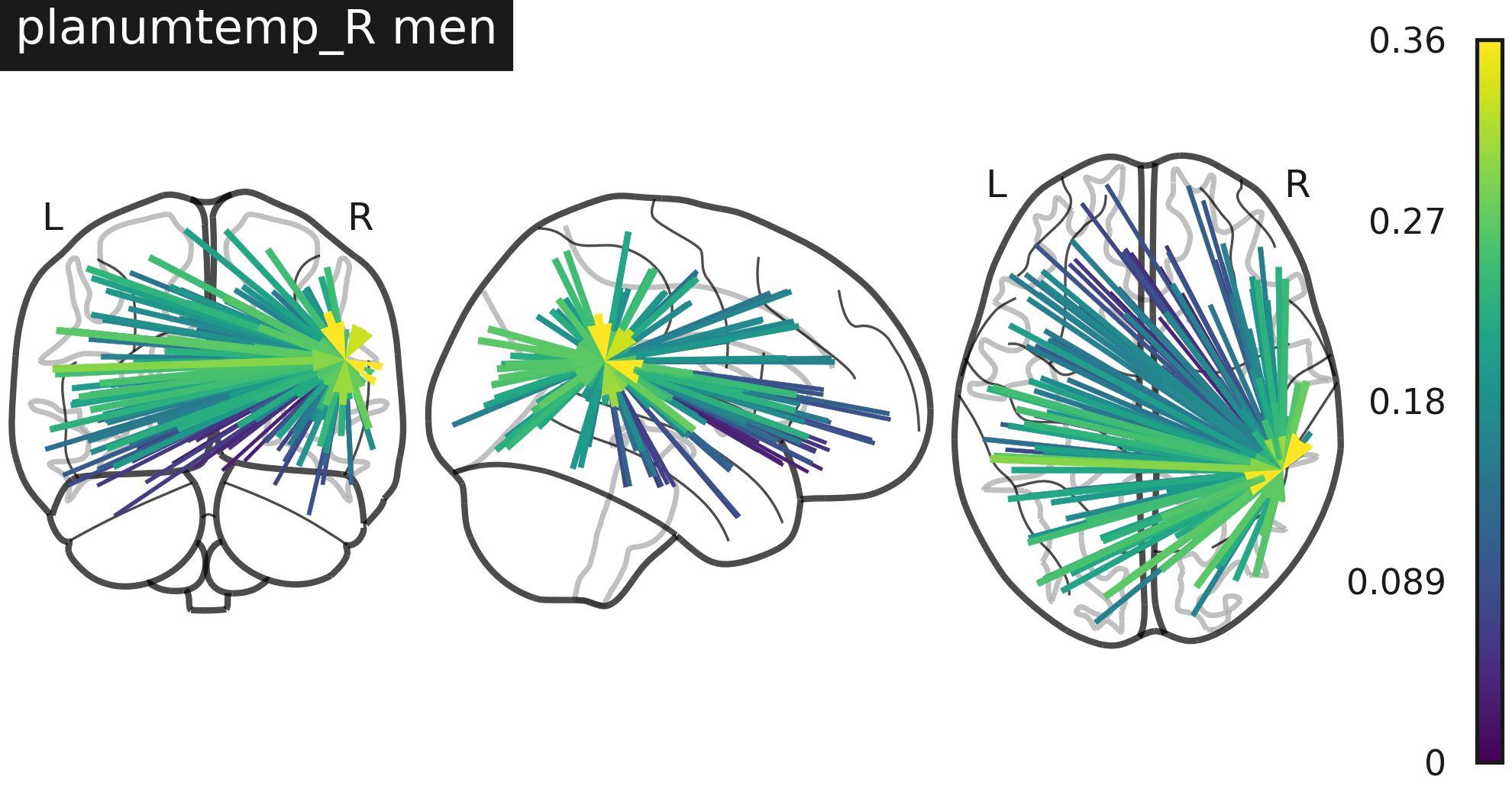


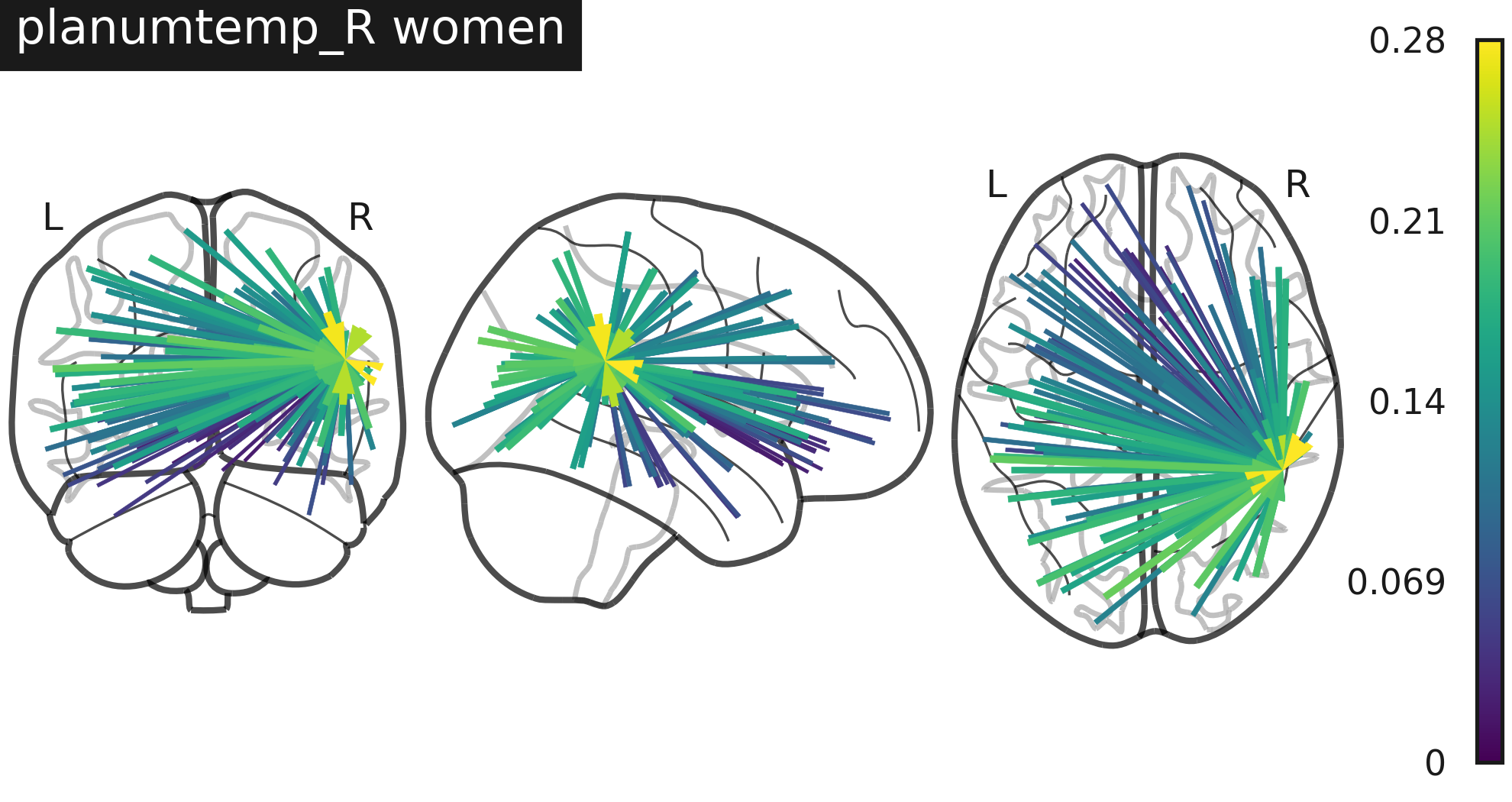

Supplement: Supplementary file 1 — Figure S1: Connectome showing the network anchored to the left aMTG in men and women. Figure S2: Connectome showing the network anchored to the right aMTG in men and women. Figure S3: Connectome showing the network anchored to the left opIFG in men and women. Figure S4: Connectome showing the network anchored to the right opIFG in men and women. Figure S5: Connectome showing the network anchored to the left pITG in men and women. Figure S6: Connectome showing the network anchored to the right pITG in men and women. Figure S7: Connectome showing the network anchored to the left planum temporale in men and women. Figure S8: Connectome showing the network anchored to the right planum temporale in men and women. [file HBM-47-e70450-s002.docx]
