# Supplementary material for: Sex Classification Based on the Functional Connectivity Patterns of the Language Network: A Resting State fMRI Study
Source: Hum Brain Mapp. 2026 Jan 10;47(1):e70450. doi: 10.1002/hbm.70450 (PMC12790092; doi:10.1002/hbm.70450)
Supplement: Supplementary file 2 — Table S1: Top 10 Discriminant Functional Connectivity Patterns Between Men and Women Anchored to the Left aMTG. Table S2: Top 10 discriminant functional connectivity patterns between men and women anchored to the right aMTG. Table S3: Top 10 discriminant functional connectivity patterns between men and women anchored to the left opIFG. Table S4: Top 10 discriminant functional connectivity patterns between men and women anchored to the right opIFG. Table S5: Top 10 discriminant functional connectivity patterns between men and women anchored to the left pITG. Table S6: Top 10 discriminant functional connectivity patterns between men and women anchored to the right pITG. Table S7: Top 10 discriminant functional connectivity patterns between men and women anchored to the left planumtemp. Table S8: Top 10 discriminant functional connectivity patterns between men and women anchored to the right planumtemp. [file HBM-47-e70450-s001.docx]

# Supplementary Tables

**TABLE 1**

*Top 10 Discriminant Functional Connectivity Patterns Between Men and Women Anchored to the Left aMTG*

| Seed ROI – Atlas parcel | Men  mean ± SD | Women  mean ± SD | *t* | *p* |
| --- | --- | --- | --- | --- |
| aMTG_L – L S_front_sup | 0.10 ± 0.08 | 0.10 ± 0.06 | 0.49 | 0.62 |
| aMTG_L – R G_pariet_inf-Angular | 0.13 ± 0.10 | 0.15 ± 0.09 | -2.17 | 0.03* |
| aMTG_L – L S_postcentral | 0.05 ± 0.10 | 0.03 ± 0.07 | 2.88 | 0.004* |
| aMTG_L – L S_intrapariet_and_P_trans | 0.03 ± 0.09 | 0.02 ± 0.06 | 1.22 | 0.22 |
| aMTG_L – R S_circular_insula_ant | 0.00 ± 0.06 | -0.00 ± 0.05 | 1.07 | 0.29 |
| aMTG_L – L G_and_S_transv_frontopol | 0.15 ± 0.08 | 0.15 ± 0.08 | 0.58 | 0.56 |
| aMTG_L – L S_orbital_lateral | 0.09 ± 0.10 | 0.08 ± 0.08 | 0.32 | 0.75 |
| aMTG_L – L G_oc-temp_med-Parahip | 0.05 ± 0.03 | 0.05 ± 0.02 | 2.26 | 0.02* |
| aMTG_L – R G_front_inf-Triangul | 0.05 ± 0.09 | 0.04 ± 0.08 | 1.11 | 0.27 |
| aMTG_L – R G_oc-temp_lat-fusifor | 0.10 ± 0.08 | 0.07 ± 0.06 | 3.99 | < 0.001* |

**TABLE 2**

*Top 10 Discriminant Functional Connectivity Patterns Between Men and Women Anchored to the Right aMTG*

| Seed ROI – Atlas parcel | Men  mean ± SD | Women  mean ± SD | *t* | *p* |
| --- | --- | --- | --- | --- |
| aMTG_R – L S_postcentral | 0.06 ± 0.10 | 0.03 ± 0.08 | 3.26 | 0.001* |
| aMTG_R – L S_front_sup | 0.11 ± 0.07 | 0.10 ± 0.06 | 1.56 | 0.12 |
| aMTG_R – L S_orbital_lateral | 0.06 ± 0.09 | 0.05 ± 0.07 | 1.17 | 0.24 |
| aMTG_R – L S_intrapariet_and_P_trans | 0.04 ± 0.09 | 0.02 ± 0.06 | 2.08 | 0.04* |
| aMTG_R – R G_pariet_inf_Angular | 0.22 ± 0.11 | 0.22 ± 0.10 | 0.20 | 0.84 |
| aMTG_R – L G_parietal_sup | 0.04 ± 0.12 | 0.00 ± 0.09 | 3.76 | 0.00* |
| aMTG_R – L S_oc-temp_med_and_Lingual | 0.09 ± 0.07 | 0.07 ± 0.06 | 3.45 | 0.001* |
| aMTG_R – L G_oc-temp_med-Parahip | 0.06 ± 0.03 | 0.05 ± 0.02 | 2.73 | 0.007* |
| aMTG_R – L S_parieto_occipital | 0.12 ± 0.09 | 0.10 ± 0.08 | 3.02 | 0.002* |
| aMTG_R – L G_and_S_transv_frontopol | 0.14 ± 0.08 | 0.13 ± 0.07 | 1.70 | 0.09 |

**TABLE 3**

*Top 10 Discriminant Functional Connectivity Patterns Between Men and Women Anchored to the Left opIFG*

| Seed ROI – Atlas parcel | Men  mean ± SD | Women  mean ± SD | *t* | *p* |
| --- | --- | --- | --- | --- |
| opIFG_L – L G_oc-temp_lat-fusifor | 0.15 ± 0.08 | 0.11 ± 0.07 | 4.66 | < 0.001* |
| opIFG_L – R S_subparietal | -0.01 ± 0.10 | -0.02 ± 0.07 | 4.34 | < 0.001* |
| opIFG_L – R G_front_inf-Orbital | 0.12 ± 0.12 | 0.09 ± 0.10 | 2.59 | 0.01* |
| opIFG_L – R Pole_occipital | 0.09 ± 0.09 | 0.08 ± 0.07 | 1.06 | 0.29 |
| opIFG_L – R S_front_inf | 0.20 ± 0.08 | 0.15 ± 0.07 | 5.88 | < 0.001* |
| opIFG_L – L Pole_occipital | 0.09 ± 0.09 | 0.09 ± 0.08 | 0.95 | 0.34 |
| opIFG_L – R G_and_S_cingul-Mid-Ant | 0.14 ± 0.08 | 0.11 ± 0.06 | 4.60 | < 0.001* |
| opIFG_L – R G_cuneus | 0.14 ± 0.10 | 0.11 ± 0.08 | 4.16 | < 0.001* |
| opIFG_L – L S_circular_insula_inf | 0.12 ± 0.08 | 0.09 ± 0.05 | 5.29 | <0.001* |
| opIFG_L – L S_intrapariet_and_P_trans | 0.27 ± 0.09 | 0.21 ± 0.09 | 6.42 | < 0.001* |

**TABLE 4**

*Top 10 Discriminant Functional Connectivity Patterns Between Men and Women Anchored to the Right opIFG*

| Seed ROI – Atlas parcel | Men  mean ± SD | Women  mean ± SD | *t* | *p* |
| --- | --- | --- | --- | --- |
| opIFG_R – L G_oc-temp_lat-fusifor | 0.16 ± 0.07 | 0.12 ± 0.06 | 5.18 | < 0.001* |
| opIFG_R – R G_and_S_cingul-Mid-Ant | 0.16 ± 0.07 | 0.13 ± 0.07 | 4.60 | < 0.001* |
| opIFG_R – L Pole_occipital | 0.11 ± 0.08 | 0.09 ± 0.07 | 2.73 | 0.007* |
| opIFG_R – R S_front_inf | 0.24 ± 0.09 | 0.19 ± 0.08 | 5.95 | < 0.001* |
| opIFG_R – R Pole_occipital | 0.10 ± 0.08 | 0.08 ± 0.07 | 2.89 | 0.004* |
| opIFG_R – L S_orbital-H_Shaped | 0.08 ± 0.05 | 0.07 ± 0.05 | 2.63 | 0.009* |
| opIFG_R – R S_circular_insula_inf | 0.11 ± 0.06 | 0.08 ± 0.05 | 5.37 | < 0.001* |
| opIFG_R – R S_subparietal | 0.02 ± 0.09 | -0.01 ± 0.06 | 4.17 | < 0.001* |
| opIFG_R – L S_circular_insula_inf | 0.10 ± 0.06 | 0.08 ± 0.04 | 5.25 | < 0.001* |
| opIFG_R – L S_oc-temp_med_and_Lingual | 0.10 ± 0.06 | 0.08 ± 0.05 | 4.83 | < 0.001* |

**TABLE 5**

*Top 10 Discriminant Functional Connectivity Patterns Between Men and Women Anchored to the Left pITG*

| Seed ROI – Atlas parcel | Men  mean ± SD | Women  mean ± SD | *t* | *p* |
| --- | --- | --- | --- | --- |
| pITG_L - L S_intrapariet_and_P_trans | 0.28 ± 0.10 | 0.25 ± 0.09 | 2.60 | 0.009 |
| pITG_L – R G_front_inf-Orbital | 0.07 ± 0.10 | 0.05 ± 0.09 | 2.18 | 0.03* |
| pITG_L - L G_cingul-Post-ventral | 0.13 ± 0.07 | 0.13 ± 0.08 | -0.77 | 0.44 |
| pITG_L - R G_cingul-Post-ventral | 0.12 ± 0.07 | 0.13 ± 0.08 | -0.61 | 0.54 |
| pITG_L – R S_front_inf | 0.18 ± 0.07 | 0.14 ± 0.06 | 3.53 | < 0.001 |
| pITG_L – L G_oc-temp_lat-fusifor | 0.14 ± 0.08 | 0.12 ± 0.07 | 3.28 | 0.001* |
| pITG_L – L G_occipital_sup | 0.13 ± 0.11 | 0.10 ± 0.10 | 3.05 | 0.002* |
| pITG_L – R Pole_occipital | 0.11 ± 0.07 | 0.10 ± 0.07 | 1.27 | 0.21 |
| pITG_L – L S_postcentral | 0.17 ± 0.09 | 0.14 ± 0.08 | 3.30 | 0.001* |
| pITG_L – L S_subparietal | 0.09 ± 0.08 | 0.09 ± 0.08 | 0.07 | 0.94 |

**TABLE 6**

*Top 10 Discriminant Functional Connectivity Patterns Between Men and Women Anchored to the Right pITG*

| Seed ROI – Atlas parcel | Men  mean ± SD | Women  mean ± SD | *t* | *p* |
| --- | --- | --- | --- | --- |
| pITG_R - L G_oc-temp_lat-fusifor | 0.20 ± 0.10 | 0.18 ± 0.09 | 2.41 | 0.02* |
| pITG_R - R G_cingul-Post-ventral | 0.14 ± 0.09 | 0.12 ± 0.09 | 2.23 | 0.03* |
| pITG_R - R S_front_inf | 0.21 ± 0.08 | 0.18 ± 0.07 | 4.01 | < 0.001* |
| pITG_R – R G_and_S_cingul-Mid-Ant | 0.12 ± 0.08 | 0.09 ± 0.06 | 4.60 | < 0.001* |
| pITG_R – L G_temp_sup-G_T_transv | 0.17 ± 0.10 | 0.13 ± 0.08 | 4.64 | < 0.001* |
| pITG_R - R G_cuneus | 0.19 ± 0.11 | 0.16 ± 0.11 | 2.72 | 0.007* |
| pITG_R – R G_oc-temp_lat-fusifor | 0.20 ± 0.10 | 0.17 ± 0.08 | 3.24 | 0.001* |
| pITG_R – R Pole_occipital | 0.14 ± 0.09 | 0.13 ± 0.08 | 1.67 | 0.09 |
| pITG_R – R S_subparietal | 0.08 ± 0.10 | 0.06 ± 0.07 | 2.74 | 0.006* |
| pITG_R – L G_occipital_sup | 0.24 ± 0.14 | 0.20 ± 0.12 | 2.77 | 0.006* |

**TABLE 7**

*Top 10 Discriminant Functional Connectivity Patterns Between Men and Women Anchored to the Left planumtemp*

| Seed ROI – Atlas parcel | Men  mean ± SD | Women  mean ± SD | *t* | *p* |
| --- | --- | --- | --- | --- |
| planumtemp_L – L G_oc-temp_lat-fusifor | 0.19 ± 0.09 | 0.17 ± 0.09 | 2.85 | 0.005* |
| planumtemp_L – R G_oc-temp_lat-fusifor | 0.18 ± 0.10 | 0.15 ± 0.09 | 3.29 | 0.001* |
| planumtemp_L - L S_oc-temp_med_and_Lingual | 0.14 ± 0.08 | 0.12 ± 0.08 | 2.74 | 0.006* |
| planumtemp_L – R G_and_S_cingul-Mid-Ant | 0.20 ± 0.10 | 0.16 ± 0.08 | 3.81 | 0.0001* |
| planumtemp_L - L S_postcentral | 0.23 ± 0.12 | 0.19 ± 0.10 | 4.06 | < 0.001* |
| planumtemp_L – L G_and_S_cingul-Mid-Ant | 0.18 ± 0.10 | 0.15 ± 0.07 | 4.07 | < 0.001* |
| planumtemp_L – R Pole_occipital | 0.15 ± 0.10 | 0.12 ± 0.09 | 2.88 | 0.004* |
| planumtemp_L - R G_temp_sup-Plan_polar | 0.13 ± 0.07 | 0.10 ± 0.06 | 4.28 | < 0.001* |
| planumtemp_L - L G_temp_sup-G_T_transv | 0.35 ± 0.15 | 0.28 ± 0.14 | 4.46 | < 0.001* |
| Planumtemp_L – R G_and_S_occipital_inf | 0.23 ± 0.12 | 0.19 ± 0.10 | 3.97 | < 0.001* |

**TABLE 8**

*Top 10 Discriminant Functional Connectivity Patterns Between Men and Women Anchored to the Right planumtemp*

| Seed ROI – Atlas parcel | Men  mean ± SD | Women  mean ± SD | *t* | *p* |
| --- | --- | --- | --- | --- |
| planumtemp_R – L S_occipital_ant | 0.25 ± 0.15 | 0.20 ± 0.12 | 3.80 | 0.0002* |
| planumtemp_R – R G_and_S_cingul-Mid-Ant | 0.17 ± 0.09 | 0.13 ± 0.07 | 5.69 | < 0.001* |
| planumtemp_R - L S_temporal_sup | 0.21 ± 0.12 | 0.17 ± 0.10 | 3.75 | 0.002* |
| planumtemp_R - L S_oc-temp_med_and_Lingual | 0.14 ± 0.08 | 0.11 ± 0.07 | 3.47 | 0.0006* |
| planumtemp_R – R S_circular_insula_inf | 0.16 ± 0.07 | 0.12 ± 0.06 | 5.52 | < 0.001* |
| planumtemp_R - R G_temp_sup-Plan_polar | 0.12 ± 0.06 | 0.09 ± 0.05 | 4.21 | < 0.001* |
| planumtemp_R – R G_oc-temp_lat-fusifor | 0.19 ± 0.10 | 0.16 ± 0.08 | 4.09 | < 0.001* |
| planumtemp_R – L S_calcarine | 0.21 ± 0.12 | 0.17 ± 0.09 | 4.18 | < 0.001* |
| planumtemp_R – L S_temporal_inf | 0.10 ± 0.07 | 0.07 ± 0.06 | 4.20 | < 0.001* |
| Planumtemp_R – L S_interm_prim-Jensen | 0.21 ± 0.14 | 0.18 ± 0.12 | 2.63 | 0.009* |
